# MicroRNA‐15a/16 regulate protein metabolism and are associated with clinical outcomes in pancreatic ductal adenocarcinoma

**DOI:** 10.14814/phy2.71015

**Published:** 2026-07-16

**Authors:** Patrick J. Ryan, Bethany C. Guerra, Peter P. Nghiem, Steven E. Riechman, Mariana Janini‐Gomes, James D. Fluckey

**Affiliations:** ^1^ Muscle Biology Laboratory, Department of Kinesiology and Sports Management Texas A&M University College Station Texas USA; ^2^ Department of Veterinary Integrative Biosciences, College of Veterinary Medicine and Biomedical Sciences Texas A&M University College Station Texas USA

**Keywords:** cancer, Metabolisim, miRNA, PDAC, proteostasis

## Abstract

Dysregulated cellular protein metabolism is a key hallmark of pancreatic ductal adenocarcinoma (PDAC). However, much remains to be learned about how this process is regulated in cancerous cells. Here we investigated the role of two co‐transcribed microRNA (miRNA) species, miR‐15a/16, in regulating cellular protein translation, cell growth, metabolic processes, and clinical features in PDAC. Using cultured PDAC models, we show that overexpression of miR‐15a/16 in cancerous cells slows proliferation and attenuates protein synthesis rates. Bioinformatics analysis reveals that these miRNAs target a broad suite of pathophysiological and metabolic pathways, including numerous genes in cancer‐ and protein‐related processes. Finally, using publicly available patient data, we report that miR‐15a/16 expression is lower in PDAC tumors and in patients with pancreatitis than in healthy controls, and that high expression of miR‐15a/16 in tumors is associated with improved survival in patients. Our results indicate that miR‐15a/16 act as regulators of protein metabolism in PDAC, with potential clinical implications for the management of this devastating disease.

## 1 INTRODUCTION

The regulation of metabolism is central to cellular health. Accordingly, aberrant cellular anabolism underlies several pathophysiological conditions, from wasting diseases to diabetes to cancer. In this last domain, there is perhaps no greater demonstration of the consequences of aberrant cell growth than in pancreatic cancer. Pancreatic ductal adenocarcinoma (PDAC) is one of the deadliest tumor types in Americans, ranking third in women and fourth in men for cancer mortality (Siegel et al., 2025). Disturbingly, PDAC cases are on the rise in the United States (Ntukidem et al., 2025), particularly in younger people (Abboud et al., 2023), with no clear explanation as to what is motivating these trends. Clearly, much remains to be discovered about the molecular drivers of carcinogenesis and progression in pancreatic cancer, emphasizing the need for investigation into these malignancies. In this vein, previous investigations have demonstrated that a broad program of metabolic reprogramming underlies cancerous behavior in PDAC, leading to the development of numerous targeted metabolic inhibitors (Raez et al., 2013). While great strides have been made in PDAC therapeutics, the ever‐adaptable nature of these tumors highlights the need for continued pursuit of new insights into their basic biology.

Reliance upon anabolic processes is one key facet of pancreatic cancer cell biology. Anabolic cellular programs are involved in numerous aspects of the cancerous phenotype, underlying features such as tumorigenesis (Bott et al., 2019), metastasis (McDonald et al., 2017), and drug resistance (Shukla et al., 2017). Although the contributions of metabolic reprogramming in features such as glucose metabolism have been well‐defined (Ying et al., 2012), less is known about how alterations in protein synthesis contribute to PDAC pathophysiology. The translation of new proteins is essential to the fundamental operations of a growing cell; indeed, the cell cycle itself cannot progress without a corresponding increase in overall protein synthesis (White‐Gilbertson et al., 2009), underlining the importance of this process to cancer cell division (Martineau, Müller, & Pyronnet, 2014). It is therefore of little surprise that cancer cells place great demands on the translational machinery (Dolfi et al., 2013; Ruggero, 2009), potentially presenting a promising therapeutic target in controlling tumor growth (Xie et al., 2024). The regulation of protein synthesis is complex, with changes in different classes of molecules such as translation factors such as eIF4E (Jia et al., 2024) or regulatory complexes such as mTOR (Jia et al., 2024) implicated in different cancer subtypes. Some investigations have documented how alterations in translation contribute to PDAC pathophysiology (Martineau, Azar, et al., 2014; Su et al., 2024), while others have found that targeting specific translation factors (Ma et al., 2019) or mTOR activity (Brown et al., 2020) suppressed PDAC growth in vitro, but much remains to be learned about the consequences of dysregulated protein synthesis in these devastating tumors.

The identification of microRNA as central regulators of cellular metabolism presents an interesting novel mechanism for global translational regulation in PDAC. MicroRNA (miRNA) are small strands of noncoding nucleic acids which serve as regulators of gene expression by binding to the 5′ untranslated region of messenger RNA strands and shepherding them for deletion in the RISC complex, a process known as RNA interference (Gebert & MacRae, 2019). Various miRNA regulate virtually every aspect of cellular metabolism (Agbu & Carthew, 2021), playing a role in processes ranging from glucose uptake (Liu et al., 2022) to calcium signaling (Du et al., 2022) in the pancreas. Accordingly, they are highly involved in PDAC pathology, being implicated as tumor promoters (Frampton et al., 2014), suppressers (Yu et al., 2010), and potential biomarkers (Schultz et al., 2014). However, to our knowledge, there have yet to be any studies of the effect of changes in miRNA species on the cellular translational machinery in PDAC, a critical intersection given the centrality of protein anabolism in PDAC pathology and the potent regulatory abilities of miRNA.

To address this gap, we elected to focus on the effects of two co‐transcribed miRNA species, miR‐15a and miR‐16 (together described as miR‐15a/16). These compounds have been shown to alter the metabolism and behavior of several tumor types, including prostate (Jin et al., 2018), gastric (Kang et al., 2015), and breast (Patel et al., 2016) cancer, and we have recently shown that they serve to suppress anabolic behavior in models of non‐small cell lung cancer (Ryan, Guerra, Nghiem, et al., 2026). Previous investigations have demonstrated that miR‐15a acts to suppress PDAC cell growth (Guo et al., 2020) and epithelial‐mesenchymal transition (Guo et al., 2014), while miR‐16 contributes to tumorigenesis (Jiao et al., 2012) and induces cachexia (Tien et al., 2024). However, no studies have evaluated the action of miR‐15a/16, which are cotranscribed from the same genetic locus (Calin et al., 2008), in PDAC. Further, little work has been done in evaluating the role of these compounds in regulating PDAC protein metabolism specifically. Therefore, to close this knowledge gap, we here set out to assess the effects of changes in both members of this miRNA family on pancreatic cancer cell growth and protein synthesis specifically, along with investigation into relevant signaling pathways and clinical outcomes associated with miR‐15a/16. Given their documented role as tumor suppressors, we hypothesized that overexpression of miR‐15a/16 would slow cellular growth and attenuate protein synthesis rates in cultured PDAC cell lines, and that these compounds would be associated with changes in the clinical course of this disease.

## 2 METHODS

### 2.1 Cell culture

We employed two pancreatic cancer cell lines, both purchased from ATCC (Manassas, VA, USA): PANC‐1 (ATCC Cat# CRL‐1469, RRID:CVCL_0480, isolated from a 56 year old White male) and MIA‐PACA‐2 (ATCC Cat# CRL‐1420, RRID:CVCL_0428, isolated from a 65 year old White male). We cultured both cell lines in DMEM (11995040; Gibco, Grand Island, NY, USA), with media supplemented with 10% fetal bovine serum (1500‐500; Avantor, Radnor, PA, USA), and 1% penicillin/streptomycin (Avantor), with cells housed in a humidified chamber maintained at 37°C and 5% CO_2_.

### 2.2 MiR15a/16 overexpression

We transfected cells with miRNA mimics to miR‐15a‐5p (4464066‐MC10235) and miR‐16‐5p (4464066‐MC10339) purchased from ThermoFisher Scientific (Waltham, MA, USA), with negative control miRNA mimic (4464058) as control. All references to miR‐15a/16 hereafter will consider the ‐5p synthesized end. We performed reverse transfection in 96‐well plates for proliferation experiments, or in 24‐well plates for protein synthesis, using Lipofectamine RNAiMAX (Thermo, 13778030) in antibiotic‐free low serum transfection medium (Transfectagro, Corning 40‐300‐CV).

### 2.3 Cell proliferation assays

We assessed cellular proliferation using a colorimetric assay (WST‐8, HY‐K0301, MedChemExpress, Monmouth Junction, NJ, USA), which produces tetrazolium salt in proportion to the number of cells in a sample well. For miRNA overexpression experiments, we reverse transfected experimental wells with mimics or control by adding cells at a density of 10,000 cells/well to transfection agents (prepared using mimics plus Lipofectamine RNAiMAX (13778150. ThermoFisher) mixed in Transfectagro transfection medium (40‐300‐CV, Corning)) and allowed to attach overnight. The next day, we replenished each well with fresh DMEM. 24 h from this point we refreshed experimental wells with media containing 10% WST‐8 assay solution, per manufacturer's instructions. We incubated the wells for 1 h, then measured absorbance at 450 nm of each assayed well using a plate reader (Spectra Max I3x, Molecular Devices, San Jose, CA, USA). Three technical replicates were performed for each well. We repeated this process in independent wells at 48 and 72 h.

### 2.4 Protein synthesis measurements

We used deuterium oxide incorporation to measure protein synthesis rates as described previously (Ryan et al., 2024). Briefly, we seeded cell suspensions at 50,000 cells/well and reverse transfected with miRNA mimics. We replenished these plates with normal media the next day, refreshing with standard media supplemented with 4% deuterium oxide (DLM‐4‐1L, Cambridge Isotope Labs Inc., Tewksbury, MA, USA) for 24 h to allow for recovery from the transfection process. Following this incorporation period, we retained a sample of media from each well, then harvested cells in lysis buffer (containing 5 mm glycerophosphate, 20 μm ATP, 25 mm HEPES, 25 mm benzamidine, 2 mm PMSF, 4 mm EDTA, 10 mm magnesium chloride, 100 mm sodium fluoride, 10 mm sodium orthovanadate, supplemented with Halt protease and phosphatase inhibitor cocktail (78437, ThermoFisher)). We then extracted cellular protein using 10% trichloroacetic acid (TCA) and centrifugation (1600 *g* for 15 min), repeated twice to collect a final pellet containing cellular protein. We subsequently used 10% hydrochloric acid and overnight incubation in a 100°C heating block with intermittent vortexing to hydrolyze the collected protein to constituent amino acids, which we derivatized using a 3:2:1 ratio solution of methyl‐8, acetone, and acetonitrile. We determined enrichment of intracellular alanine (E_A_) with gas chromatography–mass spectrometry (GCMS, Agilent 7890a GC/5975c VL MSD, Santa Clara, CA, USA). We repeated spectroscopy readings in duplicate. Separately, we isolated free deuterium from the reserved media via incubation in 10 N NaOH in 5% (vol/vol) acetone: acetonitrile for 24 h and extraction with *N*‐hexane and calculated enrichment of the cell media (E_CM_) with GCMS. Finally, we calculated cellular protein synthesis rates using the equation $\frac{E_{A}}{E_{\mathrm{CM}}\times 3.7\times t\left(h\right)\;}\times 100$, where E_A_ represents amount of protein‐bound [^2^H]alanine (mole% excess), E_CM_ is the quantity of ^2^H_2_O in cell media (mole% excess), 3.7 represents the exchange of ^2^H between cell media and alanine (e.g., 3.7 of 4 carbon‐bound hydrogen of alanine exchange with water), and *t(h)* is the duration of tracer exposure measured in hours.

### 2.5 Bioinformatics analysis

We used the DIANA‐mirRPath (Vlachos et al., 2015) version 4 web‐based analysis tool to investigate the associations between miR15a/16 and genes in the KEGG and Reactome annotated molecular pathways. DIANA‐miRPath incorporates predicted interactions from TargetScan and data from experimentally supported miRNA targets in the TarBase database to provide predicted targets for specific miRNA. Our analysis included all genes that were targeted jointly by both miR15a and miR16.

We determined miR15a/16 expression levels in PDAC using publicly available data from the Gene Expression Omnibus (GEO, available at www.ncbi.nlm.nih.gov/geo/). As it contained the largest number of tissue samples, we selected the dataset GSE24279, consisting of 136 PDAC samples, 27 pancreatitis samples, and 22 normal controls (Bauer et al., 2012).

To determine associations between miR‐15a/16 levels and clinical outcomes, we used data from PDAC patients in The Cancer Genome Atlas (TCGA), downloaded from the online Kaplan–Meier Plotter tool (Győrffy, 2024). We stratified patients by miRNA expression level (high/low), assessed through an auto‐selected best cutoff criteria which minimizes false discovery rates. This method evaluates all available cutoff values between the lower and upper quartiles of expression, then computes a Benjamini‐Hochberg corrected false discovery rate (FDR), with the lowest FDR selected as the cutoff value (Győrffy, 2023). Subsequently, we generated Kaplan–Meier curves to determine time to death or censoring.

### 2.6 Statistical analysis

We established differences in protein synthesis rates using a two‐tailed *t*‐test, while using a two‐way ANOVA (group × time) with the Sidak correction in the case of significant differences to measure changes in cellular proliferation. For bioinformatics analysis, the DIANA software includes statistical procedures based on Fisher's Exact Test, utilizing a sampling algorithm which calculates a *p* value based on predicted pathway overlap. In this case all *p* values were FDR corrected using the Benjamini‐Hochberg procedure. To determine significant differences in miR‐15a/16 expression between PDAC patients, pancreatitis patients, and healthy controls, we utilized a Wilcoxon rank‐sum test, again followed the Benjamini‐Hochberg test for FDR correction. We used a log‐rank test to determine differences in Kaplan–Meier survival curves, followed by Cox proportional hazards analysis to determine hazard ratios for patient mortality. R (R Core Team, n.d.) software was used for analysis of all experiments.

## 3 RESULTS

### 3.1 miR‐15a/16 regulate numerous cancer‐ and metabolism‐related pathways

**FIGURE 1:**
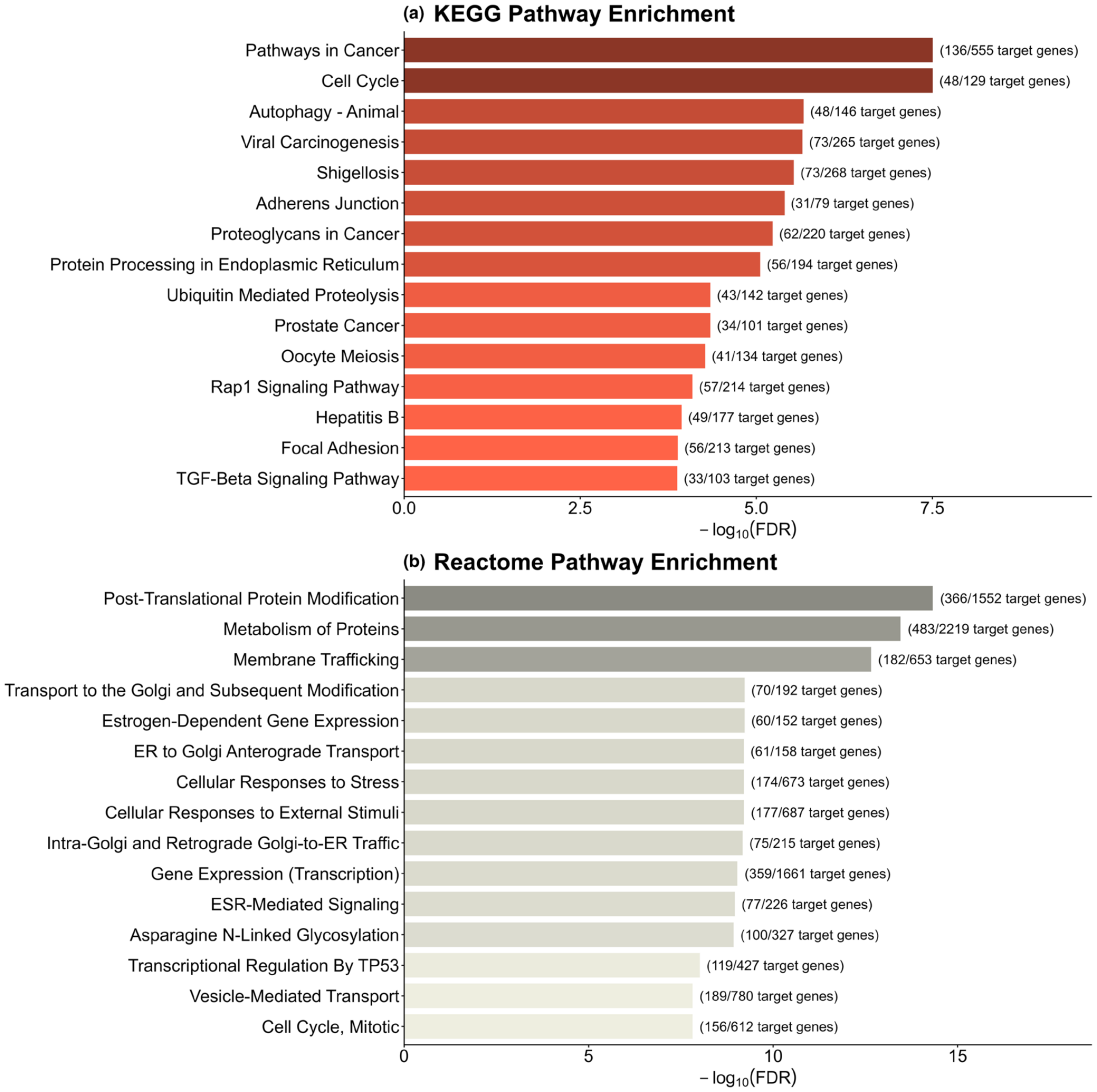
miR‐15a/16 regulate numerous cancer‐ and metabolism‐related pathways. Together, miR‐15a/16 target genes across KEGG‐annotated processes (Panel a) critical to cancer pathophysiology, including the cell cycle, cancer‐specific pathways, protein processing, and TGF‐β signaling. Further, these compounds regulate Reactome pathways (Panel b) specific to cellular protein metabolism and protein modification. Pathways are depicted as a bar chart of the −log_10_ FDR‐corrected *p* value.

**FIGURE 2:**
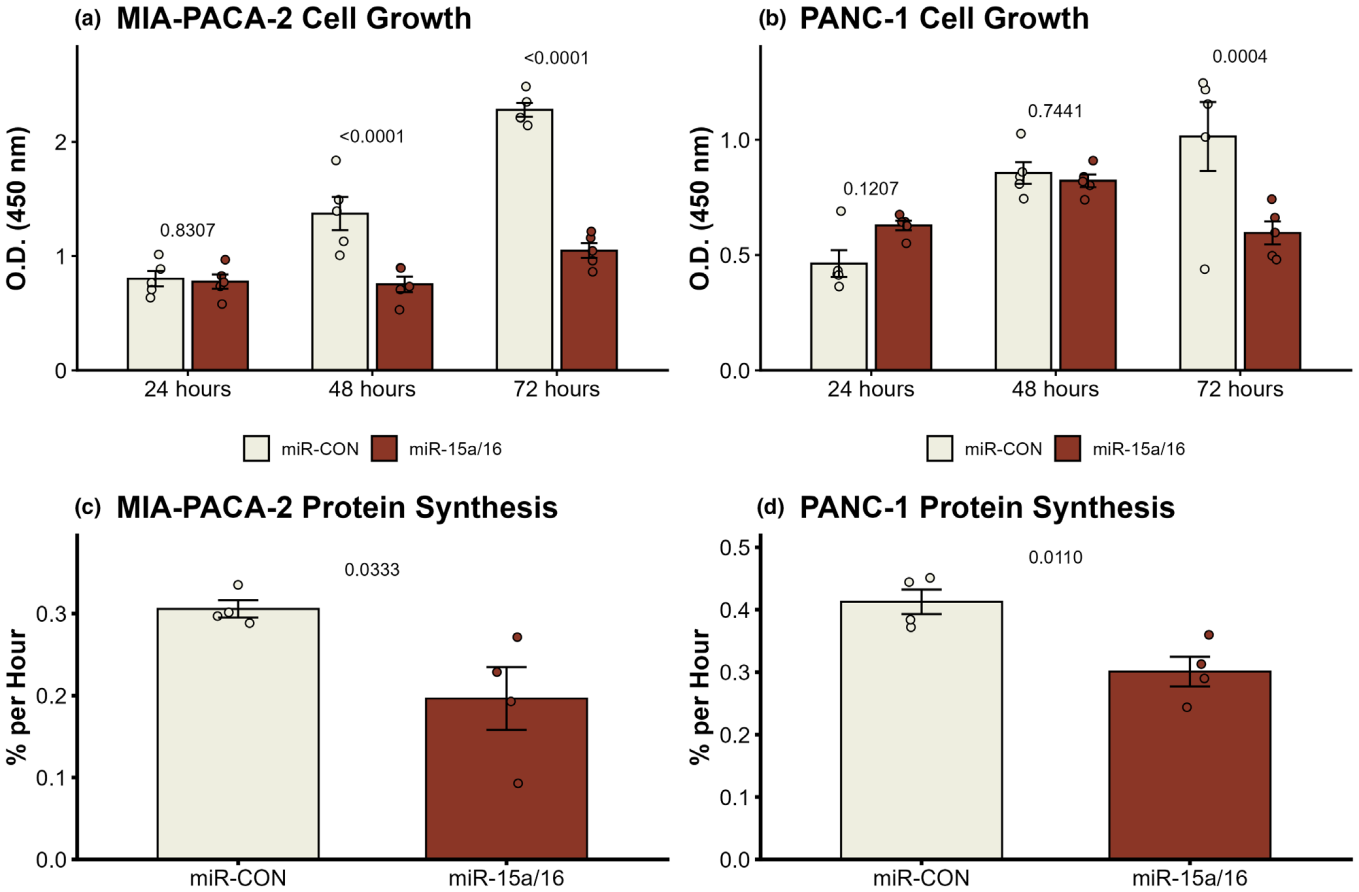
miR‐15a/16 suppress cellular proliferation and protein synthesis in PDAC. Overexpression of miR‐15a/16 mimics slows cellular growth rates in cultured PDAC cells over the course of 72 h in cultured MIA‐PACA‐2 and PANC‐1 cells (Panels a and b). MicroRNA overexpression also blunts 24‐h rates of protein synthesis in both cell lines (Panels c and d). Data are presented as means accompanied by standard errors, with individual data points depicted on each bar. For proliferation experiments (panels a and b) *n* = 5 independent experimental replicates, for protein synthesis measurements *n* = 4 independent experimental replicates. Statistical significance (*p*‐value) is displayed above each comparison.

Together, miR‐15a/16 targeted genes in nearly 100 annotated KEGG signaling pathways (a full list is available in Table S1) and 300 annotated Reactome pathways (Table S2). Within the KEGG annotation, this included cancer‐specific signaling pathways, as well as cellular processes which contribute to cancer pathophysiology, including protein handling mechanisms such as autophagy, proteolysis, and endoplasmic reticulum pathways, along with TGF‐β and RAP1 signaling (Figure 1). Reactome analysis provided a more detailed view of the cellular biology altered by miR‐15a/16, indicating that these compounds together regulated a wide swath of protein‐handling processes, including protein metabolism, post‐translational modification, and transport (Figure 2).

### 3.2 Overexpression of miR‐15a/16 suppresses cellular growth and protein synthesis in cultured PDAC cell lines

Transfection of miR‐15a/16 mimics slowed cellular proliferation by 45.2% (*p* < 0.0001) at 48% and 54.0% (*p* < 0.0001) at 72 h in MIA‐PACA‐2 cells and by 41.3% (*p* < 0.01) at 72 h in PANC‐1 cells (Figure 2), confirming that these miRNA jointly regulate PDAC cell growth. Similarly, overexpression of miR‐15a/16 mimics suppressed protein synthesis rates by 35.7% (*p* < 0.05) in MIA‐PACA‐2 cells and 27% (*p* < 0.05) in PANC‐1 cells (Figure 2), demonstrating that miR‐15a/16 serve to both regulate cell proliferation and protein anabolism.

### 3.3 miR‐15a/16 expression is lower in PDAC and pancreatitis patients than in healthy controls

**FIGURE 3:**
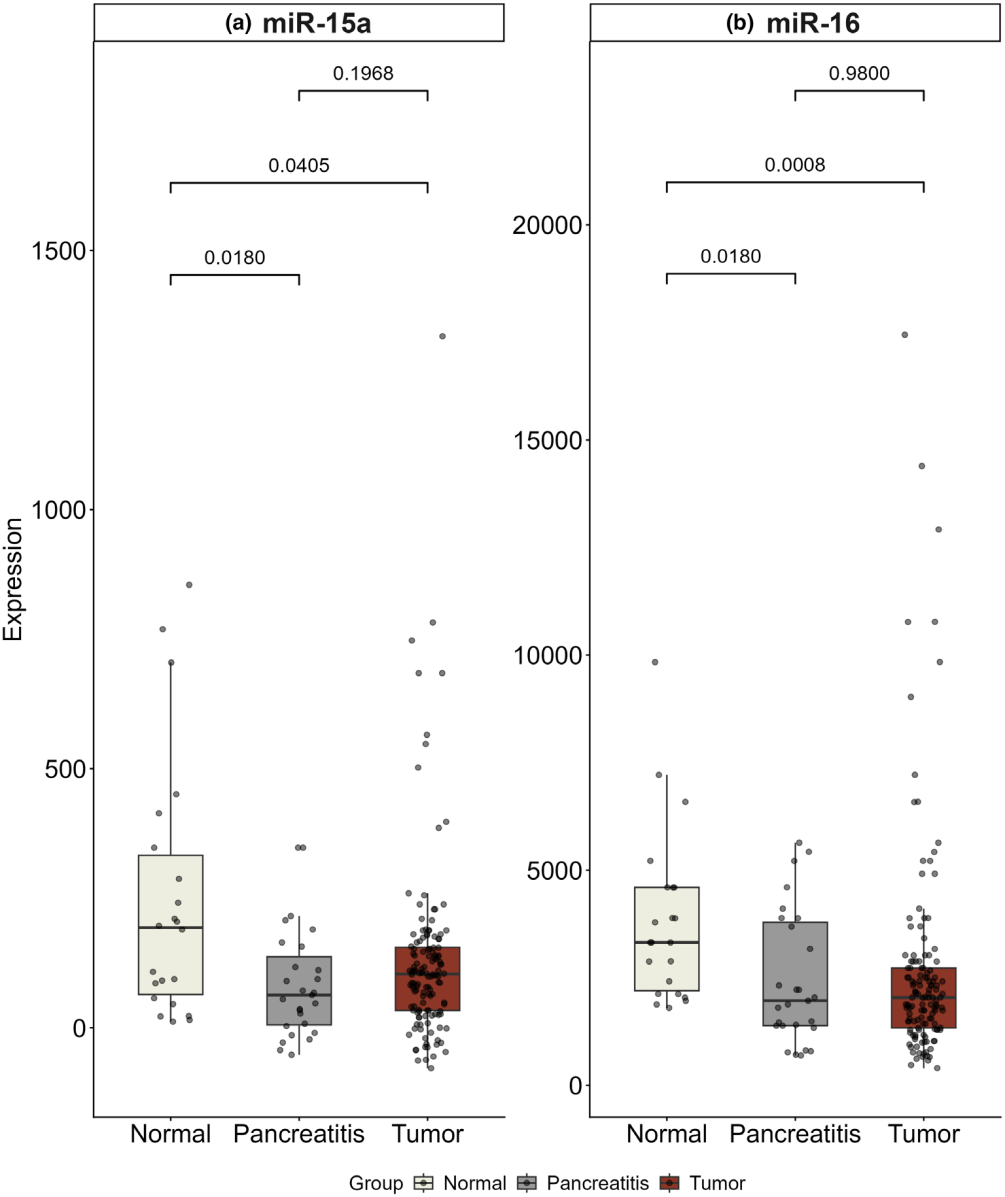
miR‐15a/16 expression in PDAC and pancreatitis. Expression of both miR‐15a (Panel a) and miR‐16 (Panel b) is lower in PDAC patient tumors (*n* = 136) than in healthy volunteer control pancreas tissue (*n* = 22). Interestingly, miR‐15a/16 is also lower in tissue isolated from pancreatitis patients (*n* = 27), a condition that may predispose to PDAC development, than in controls. Statistical significance (*p*‐value) is indicated above each comparison.

We found that miR‐15a levels were 46.1% lower (*p* < 0.05) and miR‐16 levels were 38.5% lower (*p* < 0.05) in tumor samples acquired from PDAC patients when compared to pancreatic tissue samples acquired from healthy controls (Figure 3), indicating that both miRNA species are altered in PDAC patient tumors. Additionally, miR‐15a levels were 67.2% lower (*p* < 0.05), while miR‐16 levels were 40.7% lower (*p* < 0.05) in pancreatitis patients when compared to healthy controls. While the relationship between pancreatitis and PDAC is complex, there is an increased risk of PDAC in patients with pancreatitis which worsens with pancreatitis duration (Gandhi et al., 2022). We observed no differences in miR‐15a or miR‐16 between PDAC tumors and pancreatitis patients, and although we can make no conclusions about the longitudinal relationship between these conditions, the reductions in miR‐15a/16 levels in both pancreatitis and PDAC are worthy of notice given the relationship between the two conditions.

### 3.4 Elevated levels of miR‐15a/16 are associated with clinical benefits in PDAC patients

**FIGURE 4:**
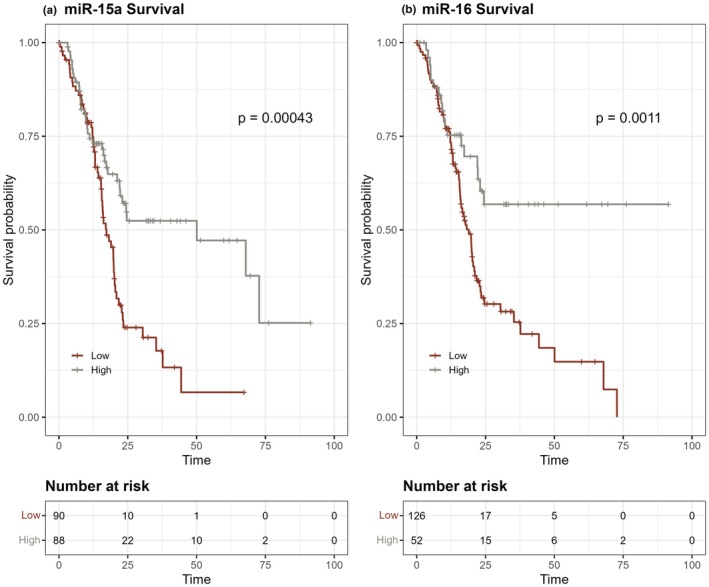
PDAC patient survival. High expression of both miR‐15a (Panel a) and miR‐16 (Panel b) is associated with improved survival in 178 patients with PDAC. Survival times are presented as Kaplan–Meier survival curves, with differences assessed and statistical significance (*p*‐value) assessed by log‐rank test.

Using TCGA data obtained from the online Kaplan–Meier Plotter web platform, we investigated the relationship between miR15a/16 levels with clinical changes in the course of disease in 178 PDAC patients (Figure 4). We found that high expression of miR15a was associated with a roughly 50% reduction in hazard of mortality (hazard ratio = 0.47 (95% CI: 0.3–0.72), *p* < 0.001) for patients and that high expression of miR16 displayed similar associations (hazard ratio = 0.43 (95% CI = 0.26–0.72), *p* < 0.005), demonstrating that miR‐15a/16 are associated with changes in the clinical course of disease for patients diagnosed with PDAC.

## 4 DISCUSSION

Altered metabolism is a central feature of PDAC. A broad suite of metabolic reprogramming events occurs in pancreatic cancer pathophysiology (Ohara et al., 2022), leading to the rapid proliferation and therapeutic resistance characteristic of these tumors. Previous studies have found that various microRNA species regulate metabolic reprogramming in PDAC, with different compounds identified as regulators of cellular glycolysis (Yang et al., 2019), autophagy (Gu et al., 2017), and arginine metabolism (Tu et al., 2020). Our findings provide new insight into the metabolic changes that occur in pancreatic cancers, indicating that miR‐15a/16 act together to control protein anabolism in these aggressive tumors. The capacity to build new proteins is critical to a growing cell, with the demands upon the synthetic machinery being accordingly increased with the rampant growth which is characteristic of a cancer (Kovalski et al., 2022). PDAC cells are no exception (Martínková et al., 2026), and prior work has shown that reduced protein synthesis results in accompanying impairments in pathophysiology, including in tumor progression and metastasis (Su et al., 2024). The finding that miR‐15a/16 can both regulate this process and suppress cell growth is thus exciting both as an insight into the fundamental etiology of and as a potential avenue for treatment in PDAC.

MicroRNAs have a tremendous impact on cellular physiology. Due to their small size, miR‐15a/16 and related molecules can target hundreds of messenger RNA transcripts and thus regulate numerous cellular signaling pathways. While we did not identify a specific mechanistic linkage behind the relationship between miR‐15a/16 and protein synthesis in these experiments, our analysis of the numerous KEGG and Reactome signaling pathways containing genes susceptible to miR‐15a/16 indicates that these compounds regulate a broad set of cellular processes, including numerous facets of protein metabolism such as protein processing and autophagy. This is especially interesting in light of work from our group and others showing that autophagic genes are required for protein synthesis and cell growth in other cancer cell types (Ryan, Guerra, Uranga, et al., 2026). Accordingly, increasing attention is being given to the contribution of various miRNA species to the process of proteostasis, the dynamic regulation of the various protein synthesizing and degrading processes within the cell (Zhang et al., 2019). For example, a recent modeling study demonstrated that tumors displaying elevations in both anabolic and catabolic activity carry worse survival outcomes for patients (Villela‐Castrejon et al., 2025), indicating the fundamental intertwining of these pathways. Proteostasis is therefore clearly critical to the growth of cancerous cells (Zhang et al., 2025), thus identifying molecules which regulate this process represents an intriguing front in combatting PDAC. While more work remains to be done, the potential for miR‐15a/16 to simultaneously target numerous cellular pathways across the broad suite of metabolic processes is an exciting avenue for future investigation of these compounds.

Additionally, our results carry several interesting clinical implications for miR‐15a/16 in PDAC. Using a previously published transcriptomic analysis (Bauer et al., 2012), we found that expression of miR‐15a/16 is lower in both PDAC tumors and pancreatic tissues acquired from pancreatitis patients when compared to healthy controls. This is especially interesting, as it suggests that alterations in these miRNA are occurring not only in PDAC, but also in unhealthy pancreas tissue which may be predisposed to clinical cancer (Ma et al., 2023). Various miRNA have long been linked to tumorigenesis in PDAC (Szafranska et al., 2007), with miR‐15a/16 identified as both tumor suppressors and potential biomarkers (Johansen et al., 2016), and our findings join those in suggesting that loss of miR‐15a/16 may drive the formation of pancreatic cancer from a pre‐clinical state by promoting cellular anabolism. However, our results imply a role for miR‐15a/16 in more than just PDAC etiology, as we found that high expression of these compounds is associated with survival in PDAC patients. These findings are in line with others which show that high miR‐15a levels are associated with clinical benefits (Guo et al., 2020), although to our knowledge we are the first to report an association between miR‐16 and PDAC patient survival. Our findings thus carry an exciting implication: that a lack of miR‐15a/16 not only contributes to PDAC formation by altering cellular protein anabolism, but that the replacement of these compounds in PDAC may provide a clinical benefit in combating these deadly tumors. Much remains to be done before this potential can be realized. However, great advancements are being made in repurposing miRNA molecules to be therapeutic tools, including the use of a miR‐16‐based therapy in an open‐label mesothelioma treatment (van Zandwijk et al., 2017). Investigation of the role microRNA, including miR‐15a/16, plays in both tumor etiology and cancer treatment remains of major importance in the biomedical sciences, and deeper understanding of the role these compounds play in PDAC pathophysiology will undoubtedly advance our ability to combat this devastating disease.

Here we demonstrate that miR‐15a/16 regulate numerous key metabolic pathways, suppress cellular proliferation, attenuate protein synthesis, exhibit reduced expression in pancreatic tumors, and are associated with improved patient survival in PDAC. Taken together, our findings indicate that these co‐transcribed miRNA species serve as central regulators of cellular protein anabolism in PDAC, capable of acting across multiple signaling pathways to exert control over cancer cell growth. In addition, we are the first to report a change in overall cellular protein synthesis rates in response to any miRNA treatment in PDAC. This discovery extends on our previous experiments in non‐small cell lung cancer (Ryan, Guerra, Nghiem, et al., 2026) where we observed a similar reduction in protein anabolism, indicating that miR‐15a/16 act as key regulators of the proteostatic machinery in malignant tumors. Finally, miR‐15a/16 are lower in both PDAC patient tumors and in patients diagnosed with pancreatitis when compared to normal healthy pancreatic tissue samples, and further, high levels of miR‐15a/16 in PDAC tumors are associated with longer survival times in patients. We conclude that miR‐15a/16 are central to the fundamental metabolic operations of PDAC, playing a key role in controlling cellular protein metabolism with implications for understanding the clinical course of this disease.

## AUTHOR CONTRIBUTIONS

**Patrick J. Ryan:** Conceptualization; data curation; formal analysis; funding acquisition; investigation; methodology; project administration; resources; validation; visualization. **Bethany C. Guerra:** Investigation. **Peter P. Nghiem:** Conceptualization; supervision. **Steven E. Riechman:** Conceptualization; formal analysis; supervision. **Mariana Janini‐Gomes:** Investigation; methodology; software. **James D. Fluckey:** Conceptualization; data curation; formal analysis; investigation; resources; supervision.

## FUNDING INFORMATION

This work was funded in part by a Department of Defense Congressionally Directed Medical Program Award. Grant Number: W81XWH‐22‐1‐0988.

## CONFLICT OF INTEREST STATEMENT

The authors have no conflicts of interest to declare in connection with this article.

## ETHICS STATEMENT

All cell culture experiments were approved by the Instiutional Biosafety Committee. No primary human data was generated during the course of these experiments.

## Supporting information


Table S1.

Table S2.

## Data Availability

All data and analytical code generated in the course of these experiments will be made available upon request to the corresponding author.

## References

[phy271015-bib-0001] Abboud, Y. , Samaan, J. S. , Oh, J. , Jiang, Y. , Randhawa, N. , Lew, D. , Ghaith, J. , Pala, P. , Leyson, C. A. , Watson, R. , Liu, Q. , Park, K. , Paski, S. , Osipov, A. , Larson, B. K. , Hendifar, A. , Atkins, K. , Nissen, N. N. , Li, D. , … Gaddam, S. (2023). Increasing pancreatic cancer incidence in young women in the United States: A population‐based time‐trend analysis, 2001–2018. Gastroenterology, 164, 978–989. 36775072 10.1053/j.gastro.2023.01.022 PMC11364483

[phy271015-bib-0002] Agbu, P. , & Carthew, R. W. (2021). MicroRNA‐mediated regulation of glucose and lipid metabolism. Nature Reviews. Molecular Cell Biology, 22, 425–438. 33772227 10.1038/s41580-021-00354-w PMC8853826

[phy271015-bib-0003] Bauer, A. S. , Keller, A. , Costello, E. , Greenhalf, W. , Bier, M. , Borries, A. , Beier, M. , Neoptolemos, J. , Büchler, M. , Werner, J. , Giese, N. , & Hoheisel, J. D. (2012). Diagnosis of pancreatic ductal adenocarcinoma and chronic pancreatitis by measurement of microRNA abundance in blood and tissue. PLoS One, 7, e34151. 22511932 10.1371/journal.pone.0034151 PMC3325244

[phy271015-bib-0004] Bott, A. J. , Shen, J. , Tonelli, C. , Zhan, L. , Sivaram, N. , Jiang, Y. P. , Yu, X. , Bhatt, V. , Chiles, E. , Zhong, H. , Maimouni, S. , Dai, W. , Velasquez, S. , Pan, J. A. , Muthalagu, N. , Morton, J. , Anthony, T. G. , Feng, H. , Lamers, W. H. , … Zong, W. X. (2019). Glutamine anabolism plays a critical role in pancreatic cancer by coupling carbon and nitrogen metabolism. Cell Reports, 29, 1287–1298. 31665640 10.1016/j.celrep.2019.09.056 PMC6886125

[phy271015-bib-0005] Brown, W. S. , McDonald, P. C. , Nemirovsky, O. , Awrey, S. , Chafe, S. C. , Schaeffer, D. F. , Li, J. , Renouf, D. J. , Stanger, B. Z. , & Dedhar, S. (2020). Overcoming adaptive resistance to KRAS and MEK inhibitors by Co‐targeting mTORC1/2 complexes in pancreatic cancer. Cell Reports Medicine, 1, 100131. 33294856 10.1016/j.xcrm.2020.100131 PMC7691443

[phy271015-bib-0006] Calin, G. A. , Cimmino, A. , Fabbri, M. , Ferracin, M. , Wojcik, S. E. , Shimizu, M. , Taccioli, C. , Zanesi, N. , Garzon, R. , Aqeilan, R. I. , Alder, H. , Volinia, S. , Rassenti, L. , Liu, X. , Liu, C. G. , Kipps, T. J. , Negrini, M. , & Croce, C. M. (2008). MiR‐15a and miR‐16‐1 cluster functions in human leukemia. Proceedings of the National Academy of Sciences, 105, 5166–5171. 10.1073/pnas.0800121105 PMC2278188 18362358

[phy271015-bib-0007] Dolfi, S. C. , Chan, L. L. Y. , Qiu, J. , Tedeschi, P. M. , Bertino, J. R. , Hirshfield, K. M. , Oltvai, Z. N. , & Vazquez, A. (2013). The metabolic demands of cancer cells are coupled to their size and protein synthesis rates. Cancer & Metabolism, 1, 20. 24279929 10.1186/2049-3002-1-20 PMC4178206

[phy271015-bib-0008] Du, W. , Liu, G. , Shi, N. , Tang, D. , Ferdek, P. E. , Jakubowska, M. A. , Liu, S. , Zhu, X. , Zhang, J. , Yao, L. , Sang, X. , Zou, S. , Liu, T. , Mukherjee, R. , Criddle, D. N. , Zheng, X. , Xia, Q. , Berggren, P. O. , Huang, W. , … Fu, X. (2022). A microRNA checkpoint for Ca^2+^ signaling and overload in acute pancreatitis. Molecular Therapy, 30, 1754–1774. 35077860 10.1016/j.ymthe.2022.01.033 PMC9077382

[phy271015-bib-0009] Frampton, A. E. , Castellano, L. , Colombo, T. , Giovannetti, E. , Krell, J. , Jacob, J. , Pellegrino, L. , Roca‐Alonso, L. , Funel, N. , Gall, T. M. H. , de Giorgio, A. , Pinho, F. G. , Fulci, V. , Britton, D. J. , Ahmad, R. , Habib, N. A. , Coombes, R. C. , Harding, V. , Knösel, T. , … Jiao, L. R. (2014). MicroRNAs cooperatively inhibit a network of tumor suppressor genes to promote pancreatic tumor growth and progression. Gastroenterology, 146, 268–277. 24120476 10.1053/j.gastro.2013.10.010

[phy271015-bib-0010] Gandhi, S. , de la Fuente, J. , Murad, M. H. , & Majumder, S. (2022). Chronic pancreatitis is a risk factor for pancreatic cancer, and incidence increases with duration of disease: A systematic review and meta‐analysis. Clinical and Translational Gastroenterology, 13, e00463. 35142721 10.14309/ctg.0000000000000463 PMC8963838

[phy271015-bib-0011] Gebert, L. F. R. , & MacRae, I. J. (2019). Regulation of microRNA function in animals. Nature Reviews. Molecular Cell Biology, 20, 21–37. 30108335 10.1038/s41580-018-0045-7 PMC6546304

[phy271015-bib-0012] Gu, D. , Jiang, M. J. , Mei, Z. , Dai, J. J. , Dai, C. Y. , Fang, C. , Huang, Q. , & Tian, L. (2017). microRNA‐7 impairs autophagy‐derived pools of glucose to suppress pancreatic cancer progression. Cancer Letters, 400, 69–78. 28450156 10.1016/j.canlet.2017.04.020

[phy271015-bib-0013] Guo, S. , Fesler, A. , Huang, W. , Wang, Y. , Yang, J. , Wang, X. , Zheng, Y. , Hwang, G. R. , Wang, H. , & Ju, J. (2020). Functional significance and therapeutic potential of miR‐15a mimic in pancreatic ductal adenocarcinoma. Molecular Therapy‐‐Nucleic Acids, 19, 228–239. 31846800 10.1016/j.omtn.2019.11.010 PMC6921186

[phy271015-bib-0014] Guo, S. , Xu, X. , Tang, Y. , Zhang, C. , Li, J. , Ouyang, Y. , Ju, J. , Bie, P. , & Wang, H. (2014). miR‐15a inhibits cell proliferation and epithelial to mesenchymal transition in pancreatic ductal adenocarcinoma by down‐regulating Bmi‐1 expression. Cancer Letters, 344, 40–46. 24252251 10.1016/j.canlet.2013.10.009

[phy271015-bib-0015] Győrffy, B. (2023). Discovery and ranking of the most robust prognostic biomarkers in serous ovarian cancer. GeroScience, 45, 1889–1898. 36856946 10.1007/s11357-023-00742-4 PMC10400493

[phy271015-bib-0016] Győrffy, B. (2024). Transcriptome‐level discovery of survival‐associated biomarkers and therapy targets in non‐small‐cell lung cancer. British Journal of Pharmacology, 181, 362–374. 37783508 10.1111/bph.16257

[phy271015-bib-0017] Jia, X. , He, X. , Huang, C. , Li, J. , Dong, Z. , & Liu, K. (2024). Protein translation: Biological processes and therapeutic strategies for human diseases. Signal Transduction and Targeted Therapy, 9, 44. 38388452 10.1038/s41392-024-01749-9 PMC10884018

[phy271015-bib-0018] Jiao, L. R. , Frampton, A. E. , Jacob, J. , Pellegrino, L. , Krell, J. , Giamas, G. , Tsim, N. , Vlavianos, P. , Cohen, P. , Ahmad, R. , Keller, A. , Habib, N. A. , Stebbing, J. , & Castellano, L. (2012). MicroRNAs targeting oncogenes are Down‐regulated in pancreatic malignant transformation from benign tumors. PLoS One, 7, e32068. 22384141 10.1371/journal.pone.0032068 PMC3284550

[phy271015-bib-0019] Jin, W. , Chen, F. , Wang, K. , Song, Y. , Fei, X. , & Wu, B. (2018). miR‐15a/miR‐16 cluster inhibits invasion of prostate cancer cells by suppressing TGF‐β signaling pathway. Biomedicine & Pharmacotherapy, 104, 637–644. 29803177 10.1016/j.biopha.2018.05.041

[phy271015-bib-0020] Johansen, J. S. , Calatayud, D. , Albieri, V. , Schultz, N. A. , Dehlendorff, C. , Werner, J. , Jensen, B. V. , Pfeiffer, P. , Bojesen, S. E. , Giese, N. , & Nielsen, K. R. (2016). The potential diagnostic value of serum microRNA signature in patients with pancreatic cancer. International Journal of Cancer, 139, 2312–2324. 10.1002/ijc.30291 27464352

[phy271015-bib-0021] Kang, W. , Tong, J. H. M. , Lung, R. W. M. , Dong, Y. , Zhao, J. , Liang, Q. , Zhang, L. , Pan, Y. , Yang, W. , Pang, J. C. S. , Cheng, A. S. L. , Yu, J. , & To, K. F. (2015). Targeting of YAP1 by microRNA‐15a and microRNA‐16‐1 exerts tumor suppressor function in gastric adenocarcinoma. Molecular Cancer, 14, 52. 25743273 10.1186/s12943-015-0323-3 PMC4342823

[phy271015-bib-0022] Kovalski, J. R. , Kuzuoglu‐Ozturk, D. , & Ruggero, D. (2022). Protein synthesis control in cancer: Selectivity and therapeutic targeting. The EMBO Journal, 41, EMBJ2021109823. 10.15252/embj.2021109823 PMC9016353 35315941

[phy271015-bib-0023] Liu, R. , Liu, C. , He, X. , Sun, P. , Zhang, B. , Yang, H. , Shi, W. , & Ruan, Q. (2022). MicroRNA‐21 promotes pancreatic β cell function through modulating glucose uptake. Nature Communications, 13, 3545. 10.1038/s41467-022-31317-0 PMC9213410 35729232

[phy271015-bib-0024] Ma, D.‐M. , Dong, X. W. , Han, X. , Ling, Z. , Lu, G. T. , Sun, Y. Y. , & Yin, X. D. (2023). Pancreatitis and pancreatic cancer risk. Technology in Cancer Research & Treatment, 22, 15330338231164875. 36972517 10.1177/15330338231164875 PMC10052482

[phy271015-bib-0025] Ma, X. , Li, B. , Liu, J. , Fu, Y. , & Luo, Y. (2019). Phosphoglycerate dehydrogenase promotes pancreatic cancer development by interacting with eIF4A1 and eIF4E. Journal of Experimental & Clinical Cancer Research, 38, 66. 30744688 10.1186/s13046-019-1053-y PMC6371491

[phy271015-bib-0026] Martineau, Y. , Azar, R. , Müller, D. , Lasfargues, C. , el Khawand, S. , Anesia, R. , Pelletier, J. , Bousquet, C. , & Pyronnet, S. (2014). Pancreatic tumours escape from translational control through 4E‐BP1 loss. Oncogene, 33, 1367–1374. 23563181 10.1038/onc.2013.100

[phy271015-bib-0027] Martineau, Y. , Müller, D. , & Pyronnet, S. (2014). Targeting protein synthesis in cancer cells. Oncoscience, 1, 484–485. 25594050 10.18632/oncoscience.63 PMC4278317

[phy271015-bib-0028] Martínková, S. , Jansová, D. , Vorel, J. , Lamačová, L. J. , Daniel, P. , Hudec, M. , Boďo, M. , Hajer, J. , Susor, A. , & Trnka, J. (2026). Ribosome transfer via tunnelling nanotubes rescues protein synthesis in pancreatic cancer cells. Cell Communication and Signaling: CCS, 24, 211. 41794726 10.1186/s12964-026-02763-w PMC13063454

[phy271015-bib-0029] McDonald, O. G. , Li, X. , Saunders, T. , Tryggvadottir, R. , Mentch, S. J. , Warmoes, M. O. , Word, A. E. , Carrer, A. , Salz, T. H. , Natsume, S. , Stauffer, K. M. , Makohon‐Moore, A. , Zhong, Y. , Wu, H. , Wellen, K. E. , Locasale, J. W. , Iacobuzio‐Donahue, C. A. , & Feinberg, A. P. (2017). Epigenomic reprogramming during pancreatic cancer progression links anabolic glucose metabolism to distant metastasis. Nature Genetics, 49, 367–376. 28092686 10.1038/ng.3753 PMC5695682

[phy271015-bib-0030] Ntukidem, O. L. , Ogedegbe, O. J. , & Bai, S. (2025). Updated trends in incidence and mortality of pancreatic cancer. An analysis of the surveillance, epidemiology, and end results (SEER) database. Journal of Clinical Oncology, 43, 786.

[phy271015-bib-0031] Ohara, Y. , Valenzuela, P. , & Hussain, S. P. (2022). The interactive role of inflammatory mediators and metabolic reprogramming in pancreatic cancer. Trends Cancer, 8, 556–569. 35525794 10.1016/j.trecan.2022.03.004 PMC9233125

[phy271015-bib-0032] Patel, N. , Garikapati, K. R. , Ramaiah, M. J. , Polavarapu, K. K. , Bhadra, U. , & Bhadra, M. P. (2016). miR‐15a/miR‐16 induces mitochondrial dependent apoptosis in breast cancer cells by suppressing oncogene BMI1. Life Sciences, 164, 60–70. 27596816 10.1016/j.lfs.2016.08.028

[phy271015-bib-0033] R Core Team R: The R Project for Statistical Computing. https://www.r‐project.org/

[phy271015-bib-0034] Raez, L. E. , Papadopoulos, K. , Ricart, A. D. , Chiorean, E. G. , DiPaola, R. S. , Stein, M. N. , Rocha Lima, C. M. , Schlesselman, J. J. , Tolba, K. , Langmuir, V. K. , Kroll, S. , Jung, D. T. , Kurtoglu, M. , Rosenblatt, J. , & Lampidis, T. J. (2013). A phase I dose‐escalation trial of 2‐deoxy‐d‐glucose alone or combined with docetaxel in patients with advanced solid tumors. Cancer Chemotherapy and Pharmacology, 71, 523–530. 23228990 10.1007/s00280-012-2045-1

[phy271015-bib-0035] Ruggero, D. (2009). The role of Myc‐induced protein synthesis in cancer. Cancer Research, 69, 8839–8843. 19934336 10.1158/0008-5472.CAN-09-1970 PMC2880919

[phy271015-bib-0036] Ryan, P. J. , Guerra, B. C. , Nghiem, P. P. , Riechman, S. E. , Janini Gomes, M. , & Fluckey, J. D. (2026). MicroRNA 15a and 16 regulate Proteostasis in non‐small cell Lung cancer. FASEB Bioadvances, 8, e70111. 42077822 10.1096/fba.2026-00075 PMC13135223

[phy271015-bib-0037] Ryan, P. J. , Guerra, B. C. , Uranga, S. , Cardin, J. M. , Riechman, S. E. , Gomes, M. J. , & Fluckey, J. D. (2026). ATG4B is required for mTORC1‐mediated anabolic activity and is associated with clinical outcomes in non‐small cell lung cancer. FEBS Open Bio, 16, 570–583. 10.1002/2211-5463.70138 PMC12955743 41065169

[phy271015-bib-0038] Ryan, P. J. , Uranga, S. , Stanelle, S. T. , Lewis, M. H. , O'Reilly, C. L. , Cardin, J. M. , Deaver, J. W. , Morton, A. B. , & Fluckey, J. D. (2024). The autophagy inhibitor NSC185058 suppresses mTORC1‐mediated protein anabolism in cultured skeletal muscle. Scientific Reports, 14, 8094. 38582781 10.1038/s41598-024-58716-1 PMC10998866

[phy271015-bib-0039] Schultz, N. A. , Dehlendorff, C. , Jensen, B. V. , Bjerregaard, J. K. , Nielsen, K. R. , Bojesen, S. E. , Calatayud, D. , Nielsen, S. E. , Yilmaz, M. , Holländer, N. H. , Andersen, K. K. , & Johansen, J. S. (2014). MicroRNA biomarkers in whole blood for detection of pancreatic cancer. JAMA, 311, 392–404. 24449318 10.1001/jama.2013.284664

[phy271015-bib-0040] Shukla, S. K. , Purohit, V. , Mehla, K. , Gunda, V. , Chaika, N. V. , Vernucci, E. , King, R. J. , Abrego, J. , Goode, G. D. , Dasgupta, A. , Illies, A. L. , Gebregiworgis, T. , Dai, B. , Augustine, J. J. , Murthy, D. , Attri, K. S. , Mashadova, O. , Grandgenett, P. M. , Powers, R. , … Singh, P. K. (2017). MUC1 and HIF‐1alpha signaling crosstalk induces anabolic glucose metabolism to impart gemcitabine resistance to pancreatic cancer. Cancer Cell, 32, 71–87. 28697344 10.1016/j.ccell.2017.06.004 PMC5533091

[phy271015-bib-0041] Siegel, R. L. , Kratzer, T. B. , Giaquinto, A. N. , Sung, H. , & Jemal, A. (2025). Cancer statistics, 2025. CA: A Cancer Journal for Clinicians, 75, 10–45. 39817679 10.3322/caac.21871 PMC11745215

[phy271015-bib-0042] Su, D. , Wang, R. , Chen, G. , Ding, C. , Liu, Y. , Tao, J. , Wang, Y. , Qiu, J. , Luo, W. , Weng, G. , Yang, G. , & Zhang, T. (2024). FBXO32 stimulates protein synthesis to drive pancreatic cancer progression and metastasis. Cancer Research, 84, 2607–2625. 38775804 10.1158/0008-5472.CAN-23-3638

[phy271015-bib-0043] Szafranska, A. E. , Davison, T. S. , John, J. , Cannon, T. , Sipos, B. , Maghnouj, A. , Labourier, E. , & Hahn, S. A. (2007). MicroRNA expression alterations are linked to tumorigenesis and non‐neoplastic processes in pancreatic ductal adenocarcinoma. Oncogene, 26, 4442–4452. 17237814 10.1038/sj.onc.1210228

[phy271015-bib-0044] Tien, S.‐C. , Chang, C. C. , Huang, C. H. , Peng, H. Y. , Chang, Y. T. , Chang, M. C. , Lee, W. H. , & Hu, C. M. (2024). Exosomal miRNA 16‐5p/29a‐3p from pancreatic cancer induce adipose atrophy by inhibiting adipogenesis and promoting lipolysis. iScience, 27, 110346. 39055920 10.1016/j.isci.2024.110346 PMC11269291

[phy271015-bib-0045] Tu, M.‐J. , Duan, Z. , Liu, Z. , Zhang, C. , Bold, R. J. , Gonzalez, F. J. , Kim, E. J. , & Yu, A. M. (2020). MicroRNA‐1291‐5p sensitizes pancreatic carcinoma cells to arginine deprivation and chemotherapy through the regulation of Arginolysis and glycolysis. Molecular Pharmacology, 98, 686–694. 33051382 10.1124/molpharm.120.000130 PMC7673485

[phy271015-bib-0046] van Zandwijk, N. , Pavlakis, N. , Kao, S. C. , Linton, A. , Boyer, M. J. , Clarke, S. , Huynh, Y. , Chrzanowska, A. , Fulham, M. J. , Bailey, D. L. , & Cooper, W. A. (2017). Safety and activity of microRNA‐loaded minicells in patients with recurrent malignant pleural mesothelioma: A first‐in‐man, phase 1, open‐label, dose‐escalation study. Lancet Oncology, 18, 1386–1396. 28870611 10.1016/S1470-2045(17)30621-6

[phy271015-bib-0047] Villela‐Castrejon, J. , Levine, H. , Kaipparettu, B. A. , Onuchic, J. N. , George, J. T. , & Jia, D. (2025). Computational modeling of cancer cell metabolism along the catabolic‐anabolic axes. Npj Systems Biology and Applications, 11, 46. 40348758 10.1038/s41540-025-00525-x PMC12065808

[phy271015-bib-0048] Vlachos, I. S. , Zagganas, K. , Paraskevopoulou, M. D. , Georgakilas, G. , Karagkouni, D. , Vergoulis, T. , Dalamagas, T. , & Hatzigeorgiou, A. G. (2015). DIANA‐miRPath v3.0: Deciphering microRNA function with experimental support. Nucleic Acids Research, 43, W460–W466. 25977294 10.1093/nar/gkv403 PMC4489228

[phy271015-bib-0049] White‐Gilbertson, S. , Kurtz, D. T. , & Voelkel‐Johnson, C. (2009). The role of protein synthesis in cell cycling and cancer. Molecular Oncology, 3, 402–408. 19546037 10.1016/j.molonc.2009.05.003 PMC2784195

[phy271015-bib-0050] Xie, B. , Zhang, M. , Li, J. , Cui, J. , Zhang, P. , Liu, F. , Wu, Y. , Deng, W. , Ma, J. , Li, X. , Pan, B. , Zhang, B. , Zhang, H. , Luo, A. , Xu, Y. , Li, M. , & Pu, Y. (2024). KAT8‐catalyzed lactylation promotes eEF1A2‐mediated protein synthesis and colorectal carcinogenesis. Proceedings of the National Academy of Sciences, 121, e2314128121. 10.1073/pnas.2314128121 PMC10895275 38359291

[phy271015-bib-0051] Yang, Y. , Ishak Gabra, M. B. , Hanse, E. A. , Lowman, X. H. , Tran, T. Q. , Li, H. , Milman, N. , Liu, J. , Reid, M. A. , Locasale, J. W. , Gil, Z. , & Kong, M. (2019). MiR‐135 suppresses glycolysis and promotes pancreatic cancer cell adaptation to metabolic stress by targeting phosphofructokinase‐1. Nature Communications, 10, 809. 10.1038/s41467-019-08759-0 PMC6379428 30778058

[phy271015-bib-0052] Ying, H. , Kimmelman, A. C. , Lyssiotis, C. A. , Hua, S. , Chu, G. C. , Fletcher‐Sananikone, E. , Locasale, J. W. , Son, J. , Zhang, H. , Coloff, J. L. , Yan, H. , Wang, W. , Chen, S. , Viale, A. , Zheng, H. , Paik, J. H. , Lim, C. , Guimaraes, A. R. , Martin, E. S. , … DePinho, R. A. (2012). Oncogenic Kras maintains pancreatic tumors through regulation of anabolic glucose metabolism. Cell, 149, 656–670. 22541435 10.1016/j.cell.2012.01.058 PMC3472002

[phy271015-bib-0053] Yu, S. , Lu, Z. , Liu, C. , Meng, Y. , Ma, Y. , Zhao, W. , Liu, J. , Yu, J. , & Chen, J. (2010). miRNA‐96 suppresses KRAS and functions as a tumor suppressor gene in pancreatic cancer. Cancer Research, 70, 6015–6025. 20610624 10.1158/0008-5472.CAN-09-4531

[phy271015-bib-0054] Zhang, C. , Li, J. , Tang, Q. , Li, L. , & Cao, D. (2025). Targeting proteostasis for cancer therapy: Current advances, challenges, and future perspectives. Molecular Cancer, 24, 265. 41121138 10.1186/s12943-025-02472-x PMC12542412

[phy271015-bib-0055] Zhang, R. , Wang, X. , Qu, J. H. , Liu, B. , Zhang, P. , Zhang, T. , Fan, P. C. , Wang, X. M. , Xiao, G. Y. , Su, Y. , Xie, Y. , Liu, Y. , Pei, J. F. , Zhang, Z. Q. , Hao, D. L. , Xu, P. , Chen, H. Z. , & Liu, D. P. (2019). Caloric restriction induces MicroRNAs to improve mitochondrial Proteostasis. iScience, 17, 155–166. 31279933 10.1016/j.isci.2019.06.028 PMC6614116

