# Supplementary material for: MicroRNA‐15a/16 regulate protein metabolism and are associated with clinical outcomes in pancreatic ductal adenocarcinoma
Source: Physiol Rep. 2026 Jul 16;14(14):e71015. doi: 10.14814/phy2.71015 (PMC13376837; doi:10.14814/phy2.71015)
Supplement: Supplementary file 1 — Table S1. [file PHY2-14-e71015-s001.docx]

Table S1. KEGG pathways targeted by miR-15a/16.

| Term Name | Term Genes | Target Genes (n) | miRNAs (n) | miRNA Names | P-value | FDR |
| --- | --- | --- | --- | --- | --- | --- |
| Pathways in cancer | 555 | 136 | 2 | hsa-miR-15a-5p,hsa-miR-16-5p | 1.83E-10 | 3.12E-08 |
| Cell cycle | 129 | 48 | 2 | hsa-miR-15a-5p,hsa-miR-16-5p | 1.65E-10 | 3.12E-08 |
| Autophagy - animal | 146 | 48 | 2 | hsa-miR-15a-5p,hsa-miR-16-5p | 1.87E-08 | 2.12E-06 |
| Viral carcinogenesis | 265 | 73 | 2 | hsa-miR-15a-5p,hsa-miR-16-5p | 2.58E-08 | 2.20E-06 |
| Shigellosis | 268 | 73 | 2 | hsa-miR-15a-5p,hsa-miR-16-5p | 4.29E-08 | 2.92E-06 |
| Adherens junction | 79 | 31 | 2 | hsa-miR-15a-5p,hsa-miR-16-5p | 6.96E-08 | 3.95E-06 |
| Proteoglycans in cancer | 220 | 62 | 2 | hsa-miR-15a-5p,hsa-miR-16-5p | 1.20E-07 | 5.86E-06 |
| Protein processing in endoplasmic reticulum | 194 | 56 | 2 | hsa-miR-15a-5p,hsa-miR-16-5p | 2.06E-07 | 8.77E-06 |
| Prostate cancer | 101 | 34 | 2 | hsa-miR-15a-5p,hsa-miR-16-5p | 1.19E-06 | 4.48E-05 |
| Ubiquitin mediated proteolysis | 142 | 43 | 2 | hsa-miR-15a-5p,hsa-miR-16-5p | 1.31E-06 | 4.48E-05 |
| Oocyte meiosis | 134 | 41 | 2 | hsa-miR-15a-5p,hsa-miR-16-5p | 1.71E-06 | 5.31E-05 |
| Rap1 signaling pathway | 214 | 57 | 2 | hsa-miR-15a-5p,hsa-miR-16-5p | 2.83E-06 | 8.04E-05 |
| Hepatitis B | 177 | 49 | 2 | hsa-miR-15a-5p,hsa-miR-16-5p | 4.39E-06 | 0.000115 |
| Focal adhesion | 213 | 56 | 2 | hsa-miR-15a-5p,hsa-miR-16-5p | 5.32E-06 | 0.00013 |
| TGF-beta signaling pathway | 103 | 33 | 2 | hsa-miR-15a-5p,hsa-miR-16-5p | 5.80E-06 | 0.000132 |
| Salmonella infection | 277 | 68 | 2 | hsa-miR-15a-5p,hsa-miR-16-5p | 7.22E-06 | 0.000154 |
| Signaling pathways regulating pluripotency of stem cells | 156 | 44 | 2 | hsa-miR-15a-5p,hsa-miR-16-5p | 7.94E-06 | 0.000159 |
| p53 signaling pathway | 75 | 26 | 2 | hsa-miR-15a-5p,hsa-miR-16-5p | 1.16E-05 | 0.00022 |
| Alcoholism | 195 | 51 | 2 | hsa-miR-15a-5p,hsa-miR-16-5p | 1.62E-05 | 0.000291 |
| Neurotrophin signaling pathway | 124 | 36 | 2 | hsa-miR-15a-5p,hsa-miR-16-5p | 2.63E-05 | 0.000448 |
| Renal cell carcinoma | 70 | 24 | 2 | hsa-miR-15a-5p,hsa-miR-16-5p | 3.08E-05 | 0.000462 |
| Hippo signaling pathway | 164 | 44 | 2 | hsa-miR-15a-5p,hsa-miR-16-5p | 3.12E-05 | 0.000462 |
| FoxO signaling pathway | 139 | 39 | 2 | hsa-miR-15a-5p,hsa-miR-16-5p | 2.92E-05 | 0.000462 |
| Human papillomavirus infection | 406 | 89 | 2 | hsa-miR-15a-5p,hsa-miR-16-5p | 3.83E-05 | 0.000544 |
| Bacterial invasion of epithelial cells | 80 | 26 | 2 | hsa-miR-15a-5p,hsa-miR-16-5p | 4.21E-05 | 0.000575 |
| MAPK signaling pathway | 329 | 74 | 2 | hsa-miR-15a-5p,hsa-miR-16-5p | 7.21E-05 | 0.000946 |
| Regulation of actin cytoskeleton | 224 | 54 | 2 | hsa-miR-15a-5p,hsa-miR-16-5p | 0.000104 | 0.001317 |
| Lysosome | 145 | 38 | 2 | hsa-miR-15a-5p,hsa-miR-16-5p | 0.00018 | 0.002193 |
| Pancreatic cancer | 78 | 24 | 2 | hsa-miR-15a-5p,hsa-miR-16-5p | 0.000214 | 0.002418 |
| TNF signaling pathway | 131 | 35 | 2 | hsa-miR-15a-5p,hsa-miR-16-5p | 0.000213 | 0.002418 |
| Lysine degradation | 69 | 22 | 2 | hsa-miR-15a-5p,hsa-miR-16-5p | 0.00022 | 0.002418 |
| Fluid shear stress and atherosclerosis | 149 | 38 | 2 | hsa-miR-15a-5p,hsa-miR-16-5p | 0.000327 | 0.003485 |
| Notch signaling pathway | 63 | 20 | 2 | hsa-miR-15a-5p,hsa-miR-16-5p | 0.000447 | 0.004617 |
| Pathogenic Escherichia coli infection | 222 | 51 | 2 | hsa-miR-15a-5p,hsa-miR-16-5p | 0.000552 | 0.005532 |
| Colorectal cancer | 88 | 25 | 2 | hsa-miR-15a-5p,hsa-miR-16-5p | 0.000618 | 0.006023 |
| Endocrine resistance | 118 | 31 | 2 | hsa-miR-15a-5p,hsa-miR-16-5p | 0.000655 | 0.006203 |
| Chronic myeloid leukemia | 79 | 23 | 2 | hsa-miR-15a-5p,hsa-miR-16-5p | 0.000692 | 0.006375 |
| Growth hormone synthesis, secretion and action | 129 | 33 | 2 | hsa-miR-15a-5p,hsa-miR-16-5p | 0.000742 | 0.006659 |
| Spinocerebellar ataxia | 145 | 36 | 2 | hsa-miR-15a-5p,hsa-miR-16-5p | 0.000796 | 0.006676 |
| PI3K-Akt signaling pathway | 372 | 77 | 2 | hsa-miR-15a-5p,hsa-miR-16-5p | 0.000803 | 0.006676 |
| Progesterone-mediated oocyte maturation | 104 | 28 | 2 | hsa-miR-15a-5p,hsa-miR-16-5p | 0.000773 | 0.006676 |
| mTOR signaling pathway | 177 | 42 | 2 | hsa-miR-15a-5p,hsa-miR-16-5p | 0.000831 | 0.006744 |
| Hepatitis C | 173 | 41 | 2 | hsa-miR-15a-5p,hsa-miR-16-5p | 0.000977 | 0.007245 |
| Melanoma | 76 | 22 | 2 | hsa-miR-15a-5p,hsa-miR-16-5p | 0.000976 | 0.007245 |
| Wnt signaling pathway | 173 | 41 | 2 | hsa-miR-15a-5p,hsa-miR-16-5p | 0.000977 | 0.007245 |
| Endocytosis | 311 | 66 | 2 | hsa-miR-15a-5p,hsa-miR-16-5p | 0.000947 | 0.007245 |
| Breast cancer | 163 | 39 | 2 | hsa-miR-15a-5p,hsa-miR-16-5p | 0.00106 | 0.007531 |
| Yersinia infection | 147 | 36 | 2 | hsa-miR-15a-5p,hsa-miR-16-5p | 0.00104 | 0.007531 |
| Various types of N-glycan biosynthesis | 40 | 14 | 2 | hsa-miR-15a-5p,hsa-miR-16-5p | 0.001085 | 0.007548 |
| Cellular senescence | 219 | 49 | 2 | hsa-miR-15a-5p,hsa-miR-16-5p | 0.001295 | 0.008725 |
| Sphingolipid signaling pathway | 133 | 33 | 2 | hsa-miR-15a-5p,hsa-miR-16-5p | 0.001305 | 0.008725 |
| Prolactin signaling pathway | 73 | 21 | 2 | hsa-miR-15a-5p,hsa-miR-16-5p | 0.001376 | 0.009021 |
| Huntington disease | 339 | 70 | 2 | hsa-miR-15a-5p,hsa-miR-16-5p | 0.001458 | 0.00938 |
| Tight junction | 182 | 42 | 2 | hsa-miR-15a-5p,hsa-miR-16-5p | 0.001491 | 0.009417 |
| Long-term depression | 64 | 19 | 2 | hsa-miR-15a-5p,hsa-miR-16-5p | 0.001523 | 0.009441 |
| Acute myeloid leukemia | 69 | 20 | 2 | hsa-miR-15a-5p,hsa-miR-16-5p | 0.001601 | 0.009752 |
| Small cell lung cancer | 100 | 26 | 2 | hsa-miR-15a-5p,hsa-miR-16-5p | 0.002024 | 0.012106 |
| Thyroid hormone signaling pathway | 137 | 33 | 2 | hsa-miR-15a-5p,hsa-miR-16-5p | 0.002208 | 0.012762 |
| Chagas disease | 116 | 29 | 2 | hsa-miR-15a-5p,hsa-miR-16-5p | 0.002205 | 0.012762 |
| cGMP-PKG signaling pathway | 175 | 40 | 2 | hsa-miR-15a-5p,hsa-miR-16-5p | 0.002299 | 0.013068 |
| Bladder cancer | 43 | 14 | 2 | hsa-miR-15a-5p,hsa-miR-16-5p | 0.00239 | 0.013361 |
| Apelin signaling pathway | 140 | 33 | 2 | hsa-miR-15a-5p,hsa-miR-16-5p | 0.003199 | 0.017596 |
| Epithelial cell signaling in Helicobacter pylori infection | 79 | 21 | 2 | hsa-miR-15a-5p,hsa-miR-16-5p | 0.003955 | 0.021408 |
| Insulin signaling pathway | 153 | 35 | 2 | hsa-miR-15a-5p,hsa-miR-16-5p | 0.004119 | 0.021947 |
| Vibrio cholerae infection | 60 | 17 | 2 | hsa-miR-15a-5p,hsa-miR-16-5p | 0.004516 | 0.023143 |
| C-type lectin receptor signaling pathway | 116 | 28 | 2 | hsa-miR-15a-5p,hsa-miR-16-5p | 0.004448 | 0.023143 |
| Dopaminergic synapse | 143 | 33 | 2 | hsa-miR-15a-5p,hsa-miR-16-5p | 0.004547 | 0.023143 |
| RNA transport | 199 | 43 | 2 | hsa-miR-15a-5p,hsa-miR-16-5p | 0.004888 | 0.024511 |
| N-Glycan biosynthesis | 51 | 15 | 2 | hsa-miR-15a-5p,hsa-miR-16-5p | 0.00508 | 0.024748 |
| Neutrophil extracellular trap formation | 205 | 44 | 2 | hsa-miR-15a-5p,hsa-miR-16-5p | 0.005059 | 0.024748 |
| Measles | 161 | 36 | 2 | hsa-miR-15a-5p,hsa-miR-16-5p | 0.005403 | 0.025672 |
| Non-small cell lung cancer | 81 | 21 | 2 | hsa-miR-15a-5p,hsa-miR-16-5p | 0.005421 | 0.025672 |
| Transcriptional misregulation in cancer | 206 | 44 | 2 | hsa-miR-15a-5p,hsa-miR-16-5p | 0.005546 | 0.025909 |
| Vasopressin-regulated water reabsorption | 47 | 14 | 2 | hsa-miR-15a-5p,hsa-miR-16-5p | 0.005909 | 0.02723 |
| Apoptosis | 151 | 34 | 2 | hsa-miR-15a-5p,hsa-miR-16-5p | 0.006018 | 0.027364 |
| Alzheimer disease | 426 | 81 | 2 | hsa-miR-15a-5p,hsa-miR-16-5p | 0.006895 | 0.030935 |
| Axon guidance | 186 | 40 | 2 | hsa-miR-15a-5p,hsa-miR-16-5p | 0.007045 | 0.031201 |
| NOD-like receptor signaling pathway | 221 | 46 | 2 | hsa-miR-15a-5p,hsa-miR-16-5p | 0.007614 | 0.032928 |
| AGE-RAGE signaling pathway in diabetic complications | 115 | 27 | 2 | hsa-miR-15a-5p,hsa-miR-16-5p | 0.007629 | 0.032928 |
| Kaposi sarcoma-associated herpesvirus infection | 245 | 50 | 2 | hsa-miR-15a-5p,hsa-miR-16-5p | 0.008219 | 0.035033 |
| Longevity regulating pathway | 105 | 25 | 2 | hsa-miR-15a-5p,hsa-miR-16-5p | 0.008331 | 0.035072 |
| Hepatocellular carcinoma | 177 | 38 | 2 | hsa-miR-15a-5p,hsa-miR-16-5p | 0.008718 | 0.036256 |
| Ras signaling pathway | 241 | 49 | 2 | hsa-miR-15a-5p,hsa-miR-16-5p | 0.009473 | 0.038455 |
| Adrenergic signaling in cardiomyocytes | 161 | 35 | 2 | hsa-miR-15a-5p,hsa-miR-16-5p | 0.009473 | 0.038455 |
| Estrogen signaling pathway | 167 | 36 | 2 | hsa-miR-15a-5p,hsa-miR-16-5p | 0.009827 | 0.039424 |
| Amyotrophic lateral sclerosis | 408 | 77 | 2 | hsa-miR-15a-5p,hsa-miR-16-5p | 0.00996 | 0.039491 |
| Gastric cancer | 162 | 35 | 2 | hsa-miR-15a-5p,hsa-miR-16-5p | 0.010431 | 0.040424 |
| Circadian rhythm | 31 | 10 | 2 | hsa-miR-15a-5p,hsa-miR-16-5p | 0.010432 | 0.040424 |
| Parathyroid hormone synthesis, secretion and action | 118 | 27 | 2 | hsa-miR-15a-5p,hsa-miR-16-5p | 0.010824 | 0.041472 |
| Pathways of neurodegeneration - multiple diseases | 539 | 98 | 2 | hsa-miR-15a-5p,hsa-miR-16-5p | 0.011572 | 0.043843 |
| AMPK signaling pathway | 130 | 29 | 2 | hsa-miR-15a-5p,hsa-miR-16-5p | 0.012089 | 0.045174 |
| Long-term potentiation | 71 | 18 | 2 | hsa-miR-15a-5p,hsa-miR-16-5p | 0.012188 | 0.045174 |
| Endometrial cancer | 61 | 16 | 2 | hsa-miR-15a-5p,hsa-miR-16-5p | 0.012653 | 0.046393 |
| Cushing syndrome | 176 | 37 | 2 | hsa-miR-15a-5p,hsa-miR-16-5p | 0.013327 | 0.047838 |
| Apoptosis - multiple species | 32 | 10 | 2 | hsa-miR-15a-5p,hsa-miR-16-5p | 0.013228 | 0.047838 |
