# Supplementary material for: MicroRNA‐15a/16 regulate protein metabolism and are associated with clinical outcomes in pancreatic ductal adenocarcinoma
Source: Physiol Rep. 2026 Jul 16;14(14):e71015. doi: 10.14814/phy2.71015 (PMC13376837; doi:10.14814/phy2.71015)
Supplement: Supplementary file 2 — Table S2. [file PHY2-14-e71015-s002.docx]

Table S2. Reactome pathways targeted by miR-15a/16

| Term Name | Term Genes | Target Genes (n) | miRNAs (n) | miRNA Names | P-value | FDR |
| --- | --- | --- | --- | --- | --- | --- |
| Post-translational protein modification | 1552 | 366 | 2 | hsa-miR-15a-5p,hsa-miR-16-5p | 1.96E-18 | 4.71E-15 |
| Metabolism of proteins | 2219 | 483 | 2 | hsa-miR-15a-5p,hsa-miR-16-5p | 2.96E-17 | 3.56E-14 |
| Membrane Trafficking | 653 | 182 | 2 | hsa-miR-15a-5p,hsa-miR-16-5p | 2.74E-16 | 2.19E-13 |
| Transport to the Golgi and subsequent modification | 192 | 70 | 2 | hsa-miR-15a-5p,hsa-miR-16-5p | 1.22E-12 | 5.87E-10 |
| Estrogen-dependent gene expression | 152 | 60 | 2 | hsa-miR-15a-5p,hsa-miR-16-5p | 9.89E-13 | 5.87E-10 |
| ER to Golgi Anterograde Transport | 158 | 61 | 2 | hsa-miR-15a-5p,hsa-miR-16-5p | 2.01E-12 | 6.14E-10 |
| Cellular responses to external stimuli | 687 | 177 | 2 | hsa-miR-15a-5p,hsa-miR-16-5p | 1.80E-12 | 6.14E-10 |
| Cellular responses to stress | 673 | 174 | 2 | hsa-miR-15a-5p,hsa-miR-16-5p | 2.05E-12 | 6.14E-10 |
| Intra-Golgi and retrograde Golgi-to-ER traffic | 215 | 75 | 2 | hsa-miR-15a-5p,hsa-miR-16-5p | 2.49E-12 | 6.63E-10 |
| Gene expression (Transcription) | 1661 | 359 | 2 | hsa-miR-15a-5p,hsa-miR-16-5p | 3.89E-12 | 9.35E-10 |
| ESR-mediated signaling | 226 | 77 | 2 | hsa-miR-15a-5p,hsa-miR-16-5p | 4.94E-12 | 1.08E-09 |
| Asparagine N-linked glycosylation | 327 | 100 | 2 | hsa-miR-15a-5p,hsa-miR-16-5p | 5.91E-12 | 1.18E-09 |
| Transcriptional Regulation by TP53 | 427 | 119 | 2 | hsa-miR-15a-5p,hsa-miR-16-5p | 5.30E-11 | 9.79E-09 |
| Vesicle-mediated transport | 780 | 189 | 2 | hsa-miR-15a-5p,hsa-miR-16-5p | 1.01E-10 | 1.51E-08 |
| Cell Cycle, Mitotic | 612 | 156 | 2 | hsa-miR-15a-5p,hsa-miR-16-5p | 9.89E-11 | 1.51E-08 |
| Chromatin modifying enzymes | 292 | 89 | 2 | hsa-miR-15a-5p,hsa-miR-16-5p | 1.07E-10 | 1.51E-08 |
| Chromatin organization | 292 | 89 | 2 | hsa-miR-15a-5p,hsa-miR-16-5p | 1.07E-10 | 1.51E-08 |
| RHO GTPase Effectors | 333 | 97 | 2 | hsa-miR-15a-5p,hsa-miR-16-5p | 2.53E-10 | 3.37E-08 |
| Cellular Senescence | 214 | 70 | 2 | hsa-miR-15a-5p,hsa-miR-16-5p | 3.65E-10 | 4.61E-08 |
| Golgi-to-ER retrograde transport | 140 | 52 | 2 | hsa-miR-15a-5p,hsa-miR-16-5p | 4.45E-10 | 5.34E-08 |
| Gene Silencing by RNA | 141 | 52 | 2 | hsa-miR-15a-5p,hsa-miR-16-5p | 5.99E-10 | 6.85E-08 |
| Regulation of cholesterol biosynthesis by SREBP (SREBF) | 57 | 29 | 2 | hsa-miR-15a-5p,hsa-miR-16-5p | 6.44E-10 | 7.03E-08 |
| Signaling by Rho GTPases | 476 | 125 | 2 | hsa-miR-15a-5p,hsa-miR-16-5p | 1.11E-09 | 1.16E-07 |
| Cell Cycle | 764 | 181 | 2 | hsa-miR-15a-5p,hsa-miR-16-5p | 1.75E-09 | 1.75E-07 |
| COPI-mediated anterograde transport | 104 | 41 | 2 | hsa-miR-15a-5p,hsa-miR-16-5p | 4.04E-09 | 3.75E-07 |
| MAP kinase activation | 64 | 30 | 2 | hsa-miR-15a-5p,hsa-miR-16-5p | 4.06E-09 | 3.75E-07 |
| Signaling by TGF-beta Receptor Complex | 75 | 33 | 2 | hsa-miR-15a-5p,hsa-miR-16-5p | 5.11E-09 | 4.54E-07 |
| Signaling by Nuclear Receptors | 318 | 90 | 2 | hsa-miR-15a-5p,hsa-miR-16-5p | 5.47E-09 | 4.69E-07 |
| Diseases of signal transduction by growth factor receptors and second messengers | 415 | 109 | 2 | hsa-miR-15a-5p,hsa-miR-16-5p | 1.32E-08 | 1.09E-06 |
| Pre-NOTCH Expression and Processing | 128 | 46 | 2 | hsa-miR-15a-5p,hsa-miR-16-5p | 1.49E-08 | 1.19E-06 |
| Transcriptional regulation by small RNAs | 109 | 41 | 2 | hsa-miR-15a-5p,hsa-miR-16-5p | 2.01E-08 | 1.51E-06 |
| Transcriptional activity of SMAD2/SMAD3:SMAD4 heterotrimer | 44 | 23 | 2 | hsa-miR-15a-5p,hsa-miR-16-5p | 1.96E-08 | 1.51E-06 |
| RNA Polymerase II Transcription | 1509 | 312 | 2 | hsa-miR-15a-5p,hsa-miR-16-5p | 2.29E-08 | 1.67E-06 |
| Oxidative Stress Induced Senescence | 134 | 47 | 2 | hsa-miR-15a-5p,hsa-miR-16-5p | 2.51E-08 | 1.77E-06 |
| Signaling by NOTCH | 275 | 78 | 2 | hsa-miR-15a-5p,hsa-miR-16-5p | 5.17E-08 | 3.55E-06 |
| Generic Transcription Pathway | 1372 | 285 | 2 | hsa-miR-15a-5p,hsa-miR-16-5p | 6.74E-08 | 4.50E-06 |
| Signaling by NTRK1 (TRKA) | 118 | 42 | 2 | hsa-miR-15a-5p,hsa-miR-16-5p | 8.56E-08 | 5.56E-06 |
| Signaling by WNT | 366 | 96 | 2 | hsa-miR-15a-5p,hsa-miR-16-5p | 1.05E-07 | 6.66E-06 |
| Interleukin-17 signaling | 72 | 30 | 2 | hsa-miR-15a-5p,hsa-miR-16-5p | 1.13E-07 | 6.89E-06 |
| Downregulation of SMAD2/3:SMAD4 transcriptional activity | 23 | 15 | 2 | hsa-miR-15a-5p,hsa-miR-16-5p | 1.15E-07 | 6.89E-06 |
| Signaling by Receptor Tyrosine Kinases | 528 | 128 | 2 | hsa-miR-15a-5p,hsa-miR-16-5p | 1.23E-07 | 7.20E-06 |
| Regulation of RUNX1 Expression and Activity | 18 | 13 | 2 | hsa-miR-15a-5p,hsa-miR-16-5p | 1.33E-07 | 7.61E-06 |
| Disease | 1819 | 361 | 2 | hsa-miR-15a-5p,hsa-miR-16-5p | 1.64E-07 | 9.15E-06 |
| TP53 Regulates Metabolic Genes | 93 | 35 | 2 | hsa-miR-15a-5p,hsa-miR-16-5p | 2.12E-07 | 1.16E-05 |
| Mitotic Prophase | 147 | 48 | 2 | hsa-miR-15a-5p,hsa-miR-16-5p | 2.23E-07 | 1.19E-05 |
| HATs acetylate histones | 148 | 48 | 2 | hsa-miR-15a-5p,hsa-miR-16-5p | 2.81E-07 | 1.46E-05 |
| Toll Like Receptor 3 (TLR3) Cascade | 103 | 37 | 2 | hsa-miR-15a-5p,hsa-miR-16-5p | 3.81E-07 | 1.90E-05 |
| Transcriptional regulation by RUNX1 | 273 | 75 | 2 | hsa-miR-15a-5p,hsa-miR-16-5p | 3.79E-07 | 1.90E-05 |
| M Phase | 462 | 113 | 2 | hsa-miR-15a-5p,hsa-miR-16-5p | 4.26E-07 | 2.09E-05 |
| Signaling by NTRKs | 137 | 45 | 2 | hsa-miR-15a-5p,hsa-miR-16-5p | 4.37E-07 | 2.10E-05 |
| Pre-NOTCH Transcription and Translation | 112 | 39 | 2 | hsa-miR-15a-5p,hsa-miR-16-5p | 4.73E-07 | 2.22E-05 |
| Signaling by TGFB family members | 104 | 37 | 2 | hsa-miR-15a-5p,hsa-miR-16-5p | 5.03E-07 | 2.27E-05 |
| MyD88 cascade initiated on plasma membrane | 92 | 34 | 2 | hsa-miR-15a-5p,hsa-miR-16-5p | 5.21E-07 | 2.27E-05 |
| Toll Like Receptor 10 (TLR10) Cascade | 92 | 34 | 2 | hsa-miR-15a-5p,hsa-miR-16-5p | 5.21E-07 | 2.27E-05 |
| Toll Like Receptor 5 (TLR5) Cascade | 92 | 34 | 2 | hsa-miR-15a-5p,hsa-miR-16-5p | 5.21E-07 | 2.27E-05 |
| COPI-dependent Golgi-to-ER retrograde traffic | 106 | 37 | 2 | hsa-miR-15a-5p,hsa-miR-16-5p | 8.63E-07 | 3.70E-05 |
| Regulation of MECP2 expression and activity | 35 | 18 | 2 | hsa-miR-15a-5p,hsa-miR-16-5p | 9.52E-07 | 4.01E-05 |
| MAPK targets/ Nuclear events mediated by MAP kinases | 32 | 17 | 2 | hsa-miR-15a-5p,hsa-miR-16-5p | 1.05E-06 | 4.36E-05 |
| MyD88-independent TLR4 cascade | 107 | 37 | 2 | hsa-miR-15a-5p,hsa-miR-16-5p | 1.12E-06 | 4.49E-05 |
| TRIF(TICAM1)-mediated TLR4 signaling | 107 | 37 | 2 | hsa-miR-15a-5p,hsa-miR-16-5p | 1.12E-06 | 4.49E-05 |
| Factors involved in megakaryocyte development and platelet production | 177 | 53 | 2 | hsa-miR-15a-5p,hsa-miR-16-5p | 1.17E-06 | 4.61E-05 |
| Positive epigenetic regulation of rRNA expression | 120 | 40 | 2 | hsa-miR-15a-5p,hsa-miR-16-5p | 1.23E-06 | 4.78E-05 |
| RUNX1 regulates genes involved in megakaryocyte differentiation and platelet function | 100 | 35 | 2 | hsa-miR-15a-5p,hsa-miR-16-5p | 1.58E-06 | 6.00E-05 |
| TRAF6 mediated induction of NFkB and MAP kinases upon TLR7/8 or 9 activation | 97 | 34 | 2 | hsa-miR-15a-5p,hsa-miR-16-5p | 2.13E-06 | 7.99E-05 |
| Small interfering RNA (siRNA) biogenesis | 9 | 8 | 2 | hsa-miR-15a-5p,hsa-miR-16-5p | 2.82E-06 | 0.000104246 |
| Activation of gene expression by SREBF (SREBP) | 44 | 20 | 2 | hsa-miR-15a-5p,hsa-miR-16-5p | 2.90E-06 | 0.000105251 |
| Circadian Clock | 70 | 27 | 2 | hsa-miR-15a-5p,hsa-miR-16-5p | 2.94E-06 | 0.000105251 |
| Activation of anterior HOX genes in hindbrain development during early embryogenesis | 124 | 40 | 2 | hsa-miR-15a-5p,hsa-miR-16-5p | 3.14E-06 | 0.000109153 |
| Activation of HOX genes during differentiation | 124 | 40 | 2 | hsa-miR-15a-5p,hsa-miR-16-5p | 3.14E-06 | 0.000109153 |
| Toll Like Receptor 9 (TLR9) Cascade | 103 | 35 | 2 | hsa-miR-15a-5p,hsa-miR-16-5p | 3.42E-06 | 0.000117448 |
| MyD88 dependent cascade initiated on endosome | 99 | 34 | 2 | hsa-miR-15a-5p,hsa-miR-16-5p | 3.60E-06 | 0.000121572 |
| Nuclear Events (kinase and transcription factor activation) | 63 | 25 | 2 | hsa-miR-15a-5p,hsa-miR-16-5p | 3.72E-06 | 0.000124038 |
| COPII-mediated vesicle transport | 71 | 27 | 2 | hsa-miR-15a-5p,hsa-miR-16-5p | 4.04E-06 | 0.00013278 |
| Toll Like Receptor 7/8 (TLR7/8) Cascade | 100 | 34 | 2 | hsa-miR-15a-5p,hsa-miR-16-5p | 4.64E-06 | 0.000150405 |
| RHO GTPases Activate Formins | 144 | 44 | 2 | hsa-miR-15a-5p,hsa-miR-16-5p | 5.18E-06 | 0.000165845 |
| Deubiquitination | 331 | 83 | 2 | hsa-miR-15a-5p,hsa-miR-16-5p | 5.30E-06 | 0.000167425 |
| Death Receptor Signalling | 154 | 46 | 2 | hsa-miR-15a-5p,hsa-miR-16-5p | 6.28E-06 | 0.000194682 |
| HCMV Early Events | 145 | 44 | 2 | hsa-miR-15a-5p,hsa-miR-16-5p | 6.32E-06 | 0.000194682 |
| Toll Like Receptor 4 (TLR4) Cascade | 137 | 42 | 2 | hsa-miR-15a-5p,hsa-miR-16-5p | 7.68E-06 | 0.000227653 |
| MyD88:MAL(TIRAP) cascade initiated on plasma membrane | 102 | 34 | 2 | hsa-miR-15a-5p,hsa-miR-16-5p | 7.59E-06 | 0.000227653 |
| Toll Like Receptor TLR6:TLR2 Cascade | 102 | 34 | 2 | hsa-miR-15a-5p,hsa-miR-16-5p | 7.59E-06 | 0.000227653 |
| HDACs deacetylate histones | 98 | 33 | 2 | hsa-miR-15a-5p,hsa-miR-16-5p | 8.06E-06 | 0.000235856 |
| HCMV Late Events | 120 | 38 | 2 | hsa-miR-15a-5p,hsa-miR-16-5p | 8.93E-06 | 0.000258373 |
| Formation of the beta-catenin:TCF transactivating complex | 95 | 32 | 2 | hsa-miR-15a-5p,hsa-miR-16-5p | 1.09E-05 | 0.000311917 |
| HCMV Infection | 171 | 49 | 2 | hsa-miR-15a-5p,hsa-miR-16-5p | 1.13E-05 | 0.000318611 |
| ERCC6 (CSB) and EHMT2 (G9a) positively regulate rRNA expression | 83 | 29 | 2 | hsa-miR-15a-5p,hsa-miR-16-5p | 1.26E-05 | 0.000350972 |
| Interactions of Rev with host cellular proteins | 37 | 17 | 2 | hsa-miR-15a-5p,hsa-miR-16-5p | 1.35E-05 | 0.00037189 |
| RORA activates gene expression | 18 | 11 | 2 | hsa-miR-15a-5p,hsa-miR-16-5p | 1.51E-05 | 0.000409652 |
| Toll Like Receptor 2 (TLR2) Cascade | 105 | 34 | 2 | hsa-miR-15a-5p,hsa-miR-16-5p | 1.54E-05 | 0.000409652 |
| Toll Like Receptor TLR1:TLR2 Cascade | 105 | 34 | 2 | hsa-miR-15a-5p,hsa-miR-16-5p | 1.54E-05 | 0.000409652 |
| Post-transcriptional silencing by small RNAs | 8 | 7 | 2 | hsa-miR-15a-5p,hsa-miR-16-5p | 1.61E-05 | 0.000423978 |
| Signaling by VEGF | 110 | 35 | 2 | hsa-miR-15a-5p,hsa-miR-16-5p | 1.77E-05 | 0.000463196 |
| Programmed Cell Death | 217 | 58 | 2 | hsa-miR-15a-5p,hsa-miR-16-5p | 1.90E-05 | 0.000490345 |
| SUMOylation | 209 | 56 | 2 | hsa-miR-15a-5p,hsa-miR-16-5p | 2.43E-05 | 0.000621481 |
| Signaling by WNT in cancer | 35 | 16 | 2 | hsa-miR-15a-5p,hsa-miR-16-5p | 2.61E-05 | 0.000660634 |
| MAPK family signaling cascades | 360 | 86 | 2 | hsa-miR-15a-5p,hsa-miR-16-5p | 2.64E-05 | 0.000660921 |
| Apoptosis | 200 | 54 | 2 | hsa-miR-15a-5p,hsa-miR-16-5p | 2.68E-05 | 0.000663422 |
| MicroRNA (miRNA) biogenesis | 25 | 13 | 2 | hsa-miR-15a-5p,hsa-miR-16-5p | 2.72E-05 | 0.000667295 |
| B-WICH complex positively regulates rRNA expression | 99 | 32 | 2 | hsa-miR-15a-5p,hsa-miR-16-5p | 2.82E-05 | 0.000683106 |
| TCF dependent signaling in response to WNT | 265 | 67 | 2 | hsa-miR-15a-5p,hsa-miR-16-5p | 3.17E-05 | 0.000759931 |
| Regulation of TP53 Activity | 182 | 50 | 2 | hsa-miR-15a-5p,hsa-miR-16-5p | 3.20E-05 | 0.000761627 |
| Transcriptional regulation of granulopoiesis | 91 | 30 | 2 | hsa-miR-15a-5p,hsa-miR-16-5p | 3.25E-05 | 0.000765898 |
| SUMOylation of ubiquitinylation proteins | 43 | 18 | 2 | hsa-miR-15a-5p,hsa-miR-16-5p | 3.61E-05 | 0.000841272 |
| DNA Damage/Telomere Stress Induced Senescence | 83 | 28 | 2 | hsa-miR-15a-5p,hsa-miR-16-5p | 3.69E-05 | 0.000850912 |
| SUMO E3 ligases SUMOylate target proteins | 203 | 54 | 2 | hsa-miR-15a-5p,hsa-miR-16-5p | 4.19E-05 | 0.000957436 |
| Regulation of PTEN gene transcription | 67 | 24 | 2 | hsa-miR-15a-5p,hsa-miR-16-5p | 4.34E-05 | 0.000983895 |
| VEGFA-VEGFR2 Pathway | 101 | 32 | 2 | hsa-miR-15a-5p,hsa-miR-16-5p | 4.40E-05 | 0.000986576 |
| Intracellular signaling by second messengers | 350 | 83 | 2 | hsa-miR-15a-5p,hsa-miR-16-5p | 4.79E-05 | 0.001065968 |
| SARS-CoV Infections | 161 | 45 | 2 | hsa-miR-15a-5p,hsa-miR-16-5p | 4.96E-05 | 0.001092219 |
| Amplification of signal from the kinetochores | 98 | 31 | 2 | hsa-miR-15a-5p,hsa-miR-16-5p | 5.95E-05 | 0.001276466 |
| Amplification of signal from unattached kinetochores via a MAD2 inhibitory signal | 98 | 31 | 2 | hsa-miR-15a-5p,hsa-miR-16-5p | 5.95E-05 | 0.001276466 |
| Formation of Senescence-Associated Heterochromatin Foci (SAHF) | 17 | 10 | 2 | hsa-miR-15a-5p,hsa-miR-16-5p | 5.95E-05 | 0.001276466 |
| Oncogene Induced Senescence | 37 | 16 | 2 | hsa-miR-15a-5p,hsa-miR-16-5p | 6.06E-05 | 0.001287932 |
| Cellular response to heat stress | 116 | 35 | 2 | hsa-miR-15a-5p,hsa-miR-16-5p | 6.17E-05 | 0.001300332 |
| Competing endogenous RNAs (ceRNAs) regulate PTEN translation | 9 | 7 | 2 | hsa-miR-15a-5p,hsa-miR-16-5p | 6.24E-05 | 0.001303506 |
| Resolution of Sister Chromatid Cohesion | 130 | 38 | 2 | hsa-miR-15a-5p,hsa-miR-16-5p | 6.57E-05 | 0.001359722 |
| SUMOylation of chromatin organization proteins | 77 | 26 | 2 | hsa-miR-15a-5p,hsa-miR-16-5p | 6.79E-05 | 0.001392916 |
| PIP3 activates AKT signaling | 307 | 74 | 2 | hsa-miR-15a-5p,hsa-miR-16-5p | 6.95E-05 | 0.001413647 |
| RNA Polymerase I Transcription | 135 | 39 | 2 | hsa-miR-15a-5p,hsa-miR-16-5p | 7.04E-05 | 0.001421066 |
| Cyclin D associated events in G1 | 49 | 19 | 2 | hsa-miR-15a-5p,hsa-miR-16-5p | 7.81E-05 | 0.001536666 |
| G1 Phase | 49 | 19 | 2 | hsa-miR-15a-5p,hsa-miR-16-5p | 7.81E-05 | 0.001536666 |
| Nuclear import of Rev protein | 34 | 15 | 2 | hsa-miR-15a-5p,hsa-miR-16-5p | 7.77E-05 | 0.001536666 |
| Toll-like Receptor Cascades | 164 | 45 | 2 | hsa-miR-15a-5p,hsa-miR-16-5p | 8.04E-05 | 0.001570266 |
| Processing of Capped Intron-Containing Pre-mRNA | 268 | 66 | 2 | hsa-miR-15a-5p,hsa-miR-16-5p | 8.55E-05 | 0.001655104 |
| Synthesis of PIPs at the ER membrane | 5 | 5 | 2 | hsa-miR-15a-5p,hsa-miR-16-5p | 9.49E-05 | 0.001822924 |
| NS1 Mediated Effects on Host Pathways | 42 | 17 | 2 | hsa-miR-15a-5p,hsa-miR-16-5p | 9.84E-05 | 0.001875049 |
| SUMOylation of DNA replication proteins | 46 | 18 | 2 | hsa-miR-15a-5p,hsa-miR-16-5p | 0.00010432 | 0.001972221 |
| PTEN Regulation | 176 | 47 | 2 | hsa-miR-15a-5p,hsa-miR-16-5p | 0.000115882 | 0.002054143 |
| Rev-mediated nuclear export of HIV RNA | 35 | 15 | 2 | hsa-miR-15a-5p,hsa-miR-16-5p | 0.000116353 | 0.002054143 |
| RAF activation | 35 | 15 | 2 | hsa-miR-15a-5p,hsa-miR-16-5p | 0.000116353 | 0.002054143 |
| Misspliced GSK3beta mutants stabilize beta-catenin | 15 | 9 | 2 | hsa-miR-15a-5p,hsa-miR-16-5p | 0.000115244 | 0.002054143 |
| phosphorylation site mutants of CTNNB1 are not targeted to the proteasome by the destruction complex | 15 | 9 | 2 | hsa-miR-15a-5p,hsa-miR-16-5p | 0.000115244 | 0.002054143 |
| S33 mutants of beta-catenin aren't phosphorylated | 15 | 9 | 2 | hsa-miR-15a-5p,hsa-miR-16-5p | 0.000115244 | 0.002054143 |
| S37 mutants of beta-catenin aren't phosphorylated | 15 | 9 | 2 | hsa-miR-15a-5p,hsa-miR-16-5p | 0.000115244 | 0.002054143 |
| S45 mutants of beta-catenin aren't phosphorylated | 15 | 9 | 2 | hsa-miR-15a-5p,hsa-miR-16-5p | 0.000115244 | 0.002054143 |
| T41 mutants of beta-catenin aren't phosphorylated | 15 | 9 | 2 | hsa-miR-15a-5p,hsa-miR-16-5p | 0.000115244 | 0.002054143 |
| ISG15 antiviral mechanism | 75 | 25 | 2 | hsa-miR-15a-5p,hsa-miR-16-5p | 0.000117907 | 0.00206638 |
| RHO GTPases activate PKNs | 97 | 30 | 2 | hsa-miR-15a-5p,hsa-miR-16-5p | 0.000123165 | 0.00214289 |
| RNA Polymerase I Promoter Clearance | 134 | 38 | 2 | hsa-miR-15a-5p,hsa-miR-16-5p | 0.000133282 | 0.002302238 |
| Metabolism of carbohydrates | 303 | 72 | 2 | hsa-miR-15a-5p,hsa-miR-16-5p | 0.000139086 | 0.002385323 |
| Infectious disease | 1054 | 208 | 2 | hsa-miR-15a-5p,hsa-miR-16-5p | 0.000142534 | 0.002427128 |
| Activation of BH3-only proteins | 32 | 14 | 2 | hsa-miR-15a-5p,hsa-miR-16-5p | 0.000150052 | 0.002434283 |
| Intrinsic Pathway for Apoptosis | 55 | 20 | 2 | hsa-miR-15a-5p,hsa-miR-16-5p | 0.000145673 | 0.002434283 |
| Negative regulation of MAPK pathway | 47 | 18 | 2 | hsa-miR-15a-5p,hsa-miR-16-5p | 0.000144495 | 0.002434283 |
| IRE1alpha activates chaperones | 51 | 19 | 2 | hsa-miR-15a-5p,hsa-miR-16-5p | 0.000146477 | 0.002434283 |
| SMAD2/SMAD3:SMAD4 heterotrimer regulates transcription | 32 | 14 | 2 | hsa-miR-15a-5p,hsa-miR-16-5p | 0.000150052 | 0.002434283 |
| NEP/NS2 Interacts with the Cellular Export Machinery | 32 | 14 | 2 | hsa-miR-15a-5p,hsa-miR-16-5p | 0.000150052 | 0.002434283 |
| Transport of Ribonucleoproteins into the Host Nucleus | 32 | 14 | 2 | hsa-miR-15a-5p,hsa-miR-16-5p | 0.000150052 | 0.002434283 |
| Epigenetic regulation of gene expression | 173 | 46 | 2 | hsa-miR-15a-5p,hsa-miR-16-5p | 0.000152071 | 0.002450479 |
| Transcriptional Regulation by MECP2 | 68 | 23 | 2 | hsa-miR-15a-5p,hsa-miR-16-5p | 0.000170222 | 0.002724684 |
| Mitotic Anaphase | 259 | 63 | 2 | hsa-miR-15a-5p,hsa-miR-16-5p | 0.00017824 | 0.002820881 |
| Activation of the AP-1 family of transcription factors | 10 | 7 | 2 | hsa-miR-15a-5p,hsa-miR-16-5p | 0.000179756 | 0.002820881 |
| RNA Polymerase I Promoter Opening | 64 | 22 | 2 | hsa-miR-15a-5p,hsa-miR-16-5p | 0.000179427 | 0.002820881 |
| Mitotic Metaphase and Anaphase | 260 | 63 | 2 | hsa-miR-15a-5p,hsa-miR-16-5p | 0.000199967 | 0.003117663 |
| Regulation of TP53 Activity through Acetylation | 33 | 14 | 2 | hsa-miR-15a-5p,hsa-miR-16-5p | 0.000223231 | 0.003435746 |
| ERKs are inactivated | 13 | 8 | 2 | hsa-miR-15a-5p,hsa-miR-16-5p | 0.000222453 | 0.003435746 |
| L1CAM interactions | 128 | 36 | 2 | hsa-miR-15a-5p,hsa-miR-16-5p | 0.000236435 | 0.003615803 |
| DARPP-32 events | 26 | 12 | 2 | hsa-miR-15a-5p,hsa-miR-16-5p | 0.00024139 | 0.003668208 |
| RHO GTPases Activate WASPs and WAVEs | 37 | 15 | 2 | hsa-miR-15a-5p,hsa-miR-16-5p | 0.000245802 | 0.00371177 |
| Oncogenic MAPK signaling | 87 | 27 | 2 | hsa-miR-15a-5p,hsa-miR-16-5p | 0.000247566 | 0.003715041 |
| Ub-specific processing proteases | 242 | 59 | 2 | hsa-miR-15a-5p,hsa-miR-16-5p | 0.000264183 | 0.003939767 |
| XBP1(S) activates chaperone genes | 49 | 18 | 2 | hsa-miR-15a-5p,hsa-miR-16-5p | 0.000267015 | 0.00395743 |
| Nervous system development | 629 | 131 | 2 | hsa-miR-15a-5p,hsa-miR-16-5p | 0.000273686 | 0.004031414 |
| Mitotic Spindle Checkpoint | 115 | 33 | 2 | hsa-miR-15a-5p,hsa-miR-16-5p | 0.000282253 | 0.004132253 |
| ERK/MAPK targets | 23 | 11 | 2 | hsa-miR-15a-5p,hsa-miR-16-5p | 0.000298486 | 0.004343424 |
| Regulation of HSF1-mediated heat shock response | 97 | 29 | 2 | hsa-miR-15a-5p,hsa-miR-16-5p | 0.000303367 | 0.004387851 |
| Role of ABL in ROBO-SLIT signaling | 8 | 6 | 2 | hsa-miR-15a-5p,hsa-miR-16-5p | 0.00031216 | 0.004487998 |
| Vpr-mediated nuclear import of PICs | 34 | 14 | 2 | hsa-miR-15a-5p,hsa-miR-16-5p | 0.000325109 | 0.004646348 |
| EML4 and NUDC in mitotic spindle formation | 121 | 34 | 2 | hsa-miR-15a-5p,hsa-miR-16-5p | 0.000354314 | 0.005033777 |
| Regulation of TP53 Expression and Degradation | 42 | 16 | 2 | hsa-miR-15a-5p,hsa-miR-16-5p | 0.000359218 | 0.005073422 |
| Glycosaminoglycan metabolism | 126 | 35 | 2 | hsa-miR-15a-5p,hsa-miR-16-5p | 0.000371059 | 0.005204282 |
| G0 and Early G1 | 27 | 12 | 2 | hsa-miR-15a-5p,hsa-miR-16-5p | 0.000372818 | 0.005204282 |
| Beta-catenin phosphorylation cascade | 17 | 9 | 2 | hsa-miR-15a-5p,hsa-miR-16-5p | 0.000414796 | 0.005756788 |
| Defective EXT1 causes exostoses 1, TRPS2 and CHDS | 14 | 8 | 2 | hsa-miR-15a-5p,hsa-miR-16-5p | 0.000447627 | 0.005841041 |
| Defective EXT2 causes exostoses 2 | 14 | 8 | 2 | hsa-miR-15a-5p,hsa-miR-16-5p | 0.000447627 | 0.005841041 |
| AMER1 mutants destabilize the destruction complex | 14 | 8 | 2 | hsa-miR-15a-5p,hsa-miR-16-5p | 0.000447627 | 0.005841041 |
| APC truncation mutants have impaired AXIN binding | 14 | 8 | 2 | hsa-miR-15a-5p,hsa-miR-16-5p | 0.000447627 | 0.005841041 |
| AXIN missense mutants destabilize the destruction complex | 14 | 8 | 2 | hsa-miR-15a-5p,hsa-miR-16-5p | 0.000447627 | 0.005841041 |
| AXIN mutants destabilize the destruction complex, activating WNT signaling | 14 | 8 | 2 | hsa-miR-15a-5p,hsa-miR-16-5p | 0.000447627 | 0.005841041 |
| truncated APC mutants destabilize the destruction complex | 14 | 8 | 2 | hsa-miR-15a-5p,hsa-miR-16-5p | 0.000447627 | 0.005841041 |
| Truncations of AMER1 destabilize the destruction complex | 14 | 8 | 2 | hsa-miR-15a-5p,hsa-miR-16-5p | 0.000447627 | 0.005841041 |
| RHO GTPases activate KTN1 | 11 | 7 | 2 | hsa-miR-15a-5p,hsa-miR-16-5p | 0.000427151 | 0.005841041 |
| Regulation of PTEN mRNA translation | 11 | 7 | 2 | hsa-miR-15a-5p,hsa-miR-16-5p | 0.000427151 | 0.005841041 |
| Prolonged ERK activation events | 14 | 8 | 2 | hsa-miR-15a-5p,hsa-miR-16-5p | 0.000447627 | 0.005841041 |
| Activated PKN1 stimulates transcription of AR (androgen receptor) regulated genes KLK2 and KLK3 | 68 | 22 | 2 | hsa-miR-15a-5p,hsa-miR-16-5p | 0.000475754 | 0.006174509 |
| Cell Cycle Checkpoints | 326 | 74 | 2 | hsa-miR-15a-5p,hsa-miR-16-5p | 0.000493497 | 0.006370356 |
| Signaling by NOTCH1 | 77 | 24 | 2 | hsa-miR-15a-5p,hsa-miR-16-5p | 0.000499094 | 0.006408151 |
| Ca2+ pathway | 64 | 21 | 2 | hsa-miR-15a-5p,hsa-miR-16-5p | 0.00051326 | 0.006554979 |
| Senescence-Associated Secretory Phenotype (SASP) | 119 | 33 | 2 | hsa-miR-15a-5p,hsa-miR-16-5p | 0.00055614 | 0.007065037 |
| mRNA Splicing | 208 | 51 | 2 | hsa-miR-15a-5p,hsa-miR-16-5p | 0.000580172 | 0.007331542 |
| Glucose metabolism | 96 | 28 | 2 | hsa-miR-15a-5p,hsa-miR-16-5p | 0.000595045 | 0.007480124 |
| Nuclear Envelope (NE) Reassembly | 78 | 24 | 2 | hsa-miR-15a-5p,hsa-miR-16-5p | 0.000615315 | 0.007694639 |
| Viral Messenger RNA Synthesis | 44 | 16 | 2 | hsa-miR-15a-5p,hsa-miR-16-5p | 0.000658629 | 0.008151385 |
| Association of TriC/CCT with target proteins during biosynthesis | 44 | 16 | 2 | hsa-miR-15a-5p,hsa-miR-16-5p | 0.000658629 | 0.008151385 |
| Antiviral mechanism by IFN-stimulated genes | 83 | 25 | 2 | hsa-miR-15a-5p,hsa-miR-16-5p | 0.000680722 | 0.008381609 |
| Constitutive Signaling by NOTCH1 HD+PEST Domain Mutants | 61 | 20 | 2 | hsa-miR-15a-5p,hsa-miR-16-5p | 0.000699503 | 0.008397539 |
| Constitutive Signaling by NOTCH1 PEST Domain Mutants | 61 | 20 | 2 | hsa-miR-15a-5p,hsa-miR-16-5p | 0.000699503 | 0.008397539 |
| Signaling by NOTCH1 HD+PEST Domain Mutants in Cancer | 61 | 20 | 2 | hsa-miR-15a-5p,hsa-miR-16-5p | 0.000699503 | 0.008397539 |
| Signaling by NOTCH1 in Cancer | 61 | 20 | 2 | hsa-miR-15a-5p,hsa-miR-16-5p | 0.000699503 | 0.008397539 |
| Signaling by NOTCH1 PEST Domain Mutants in Cancer | 61 | 20 | 2 | hsa-miR-15a-5p,hsa-miR-16-5p | 0.000699503 | 0.008397539 |
| Aberrant regulation of mitotic G1/S transition in cancer due to RB1 defects | 18 | 9 | 2 | hsa-miR-15a-5p,hsa-miR-16-5p | 0.000714466 | 0.008492237 |
| Defective binding of RB1 mutants to E2F1,(E2F2, E2F3) | 18 | 9 | 2 | hsa-miR-15a-5p,hsa-miR-16-5p | 0.000714466 | 0.008492237 |
| mRNA Splicing - Major Pathway | 200 | 49 | 2 | hsa-miR-15a-5p,hsa-miR-16-5p | 0.000749607 | 0.008866043 |
| SUMOylation of transcription cofactors | 53 | 18 | 2 | hsa-miR-15a-5p,hsa-miR-16-5p | 0.000798466 | 0.009351788 |
| SUMOylation of RNA binding proteins | 53 | 18 | 2 | hsa-miR-15a-5p,hsa-miR-16-5p | 0.000798466 | 0.009351788 |
| EGR2 and SOX10-mediated initiation of Schwann cell myelination | 29 | 12 | 2 | hsa-miR-15a-5p,hsa-miR-16-5p | 0.000819928 | 0.009556539 |
| PTK6 Regulates RHO GTPases, RAS GTPase and MAP kinases | 15 | 8 | 2 | hsa-miR-15a-5p,hsa-miR-16-5p | 0.000827493 | 0.009598119 |
| PRC2 methylates histones and DNA | 75 | 23 | 2 | hsa-miR-15a-5p,hsa-miR-16-5p | 0.000834044 | 0.009627598 |
| Signaling by Interleukins | 512 | 107 | 2 | hsa-miR-15a-5p,hsa-miR-16-5p | 0.000857588 | 0.009852008 |
| p75 NTR receptor-mediated signalling | 103 | 29 | 2 | hsa-miR-15a-5p,hsa-miR-16-5p | 0.000899506 | 0.010092121 |
| Interactions of Vpr with host cellular proteins | 37 | 14 | 2 | hsa-miR-15a-5p,hsa-miR-16-5p | 0.000897781 | 0.010092121 |
| Regulation of TP53 Degradation | 41 | 15 | 2 | hsa-miR-15a-5p,hsa-miR-16-5p | 0.000895665 | 0.010092121 |
| Export of Viral Ribonucleoproteins from Nucleus | 37 | 14 | 2 | hsa-miR-15a-5p,hsa-miR-16-5p | 0.000897781 | 0.010092121 |
| Frs2-mediated activation | 12 | 7 | 2 | hsa-miR-15a-5p,hsa-miR-16-5p | 0.000886216 | 0.010092121 |
| Signaling by Hippo | 22 | 10 | 2 | hsa-miR-15a-5p,hsa-miR-16-5p | 0.000930115 | 0.010338914 |
| HS-GAG degradation | 22 | 10 | 2 | hsa-miR-15a-5p,hsa-miR-16-5p | 0.000930115 | 0.010338914 |
| Unfolded Protein Response (UPR) | 94 | 27 | 2 | hsa-miR-15a-5p,hsa-miR-16-5p | 0.00095677 | 0.010537642 |
| TP53 Regulates Transcription of Cell Cycle Genes | 58 | 19 | 2 | hsa-miR-15a-5p,hsa-miR-16-5p | 0.000954183 | 0.010537642 |
| Autophagy | 162 | 41 | 2 | hsa-miR-15a-5p,hsa-miR-16-5p | 0.000996377 | 0.010923748 |
| Recycling pathway of L1 | 50 | 17 | 2 | hsa-miR-15a-5p,hsa-miR-16-5p | 0.001090681 | 0.011903291 |
| Protein ubiquitination | 81 | 24 | 2 | hsa-miR-15a-5p,hsa-miR-16-5p | 0.001116234 | 0.012127052 |
| Diseases of programmed cell death | 30 | 12 | 2 | hsa-miR-15a-5p,hsa-miR-16-5p | 0.001173315 | 0.012689768 |
| HSP90 chaperone cycle for steroid hormone receptors (SHR) | 68 | 21 | 2 | hsa-miR-15a-5p,hsa-miR-16-5p | 0.001248835 | 0.013445979 |
| SUMOylation of DNA damage response and repair proteins | 91 | 26 | 2 | hsa-miR-15a-5p,hsa-miR-16-5p | 0.001282748 | 0.013749456 |
| Apoptotic execution phase | 55 | 18 | 2 | hsa-miR-15a-5p,hsa-miR-16-5p | 0.001302895 | 0.013903334 |
| Chondroitin sulfate/dermatan sulfate metabolism | 51 | 17 | 2 | hsa-miR-15a-5p,hsa-miR-16-5p | 0.001400339 | 0.014877051 |
| Deregulated CDK5 triggers multiple neurodegenerative pathways in Alzheimer's disease models | 23 | 10 | 2 | hsa-miR-15a-5p,hsa-miR-16-5p | 0.001415983 | 0.014911295 |
| Neurodegenerative Diseases | 23 | 10 | 2 | hsa-miR-15a-5p,hsa-miR-16-5p | 0.001415983 | 0.014911295 |
| Sema3A PAK dependent Axon repulsion | 16 | 8 | 2 | hsa-miR-15a-5p,hsa-miR-16-5p | 0.001428265 | 0.014974952 |
| PP2A-mediated dephosphorylation of key metabolic factors | 7 | 5 | 2 | hsa-miR-15a-5p,hsa-miR-16-5p | 0.001507624 | 0.015738281 |
| Post-translational protein phosphorylation | 111 | 30 | 2 | hsa-miR-15a-5p,hsa-miR-16-5p | 0.001519121 | 0.015789649 |
| SIRT1 negatively regulates rRNA expression | 69 | 21 | 2 | hsa-miR-15a-5p,hsa-miR-16-5p | 0.001534253 | 0.015878195 |
| Downregulation of TGF-beta receptor signaling | 27 | 11 | 2 | hsa-miR-15a-5p,hsa-miR-16-5p | 0.001569013 | 0.016099146 |
| Postmitotic nuclear pore complex (NPC) reformation | 27 | 11 | 2 | hsa-miR-15a-5p,hsa-miR-16-5p | 0.001569013 | 0.016099146 |
| Organelle biogenesis and maintenance | 318 | 70 | 2 | hsa-miR-15a-5p,hsa-miR-16-5p | 0.001645789 | 0.016815061 |
| SUMOylation of SUMOylation proteins | 35 | 13 | 2 | hsa-miR-15a-5p,hsa-miR-16-5p | 0.001658996 | 0.016823276 |
| Mitotic Telophase/Cytokinesis | 13 | 7 | 2 | hsa-miR-15a-5p,hsa-miR-16-5p | 0.001660607 | 0.016823276 |
| Glycolysis | 74 | 22 | 2 | hsa-miR-15a-5p,hsa-miR-16-5p | 0.001681605 | 0.016964428 |
| Folding of actin by CCT/TriC | 10 | 6 | 2 | hsa-miR-15a-5p,hsa-miR-16-5p | 0.001759396 | 0.017601287 |
| MASTL Facilitates Mitotic Progression | 10 | 6 | 2 | hsa-miR-15a-5p,hsa-miR-16-5p | 0.001759396 | 0.017601287 |
| SUMOylation of transcription factors | 20 | 9 | 2 | hsa-miR-15a-5p,hsa-miR-16-5p | 0.001832404 | 0.018255608 |
| Interleukin-12 family signaling | 61 | 19 | 2 | hsa-miR-15a-5p,hsa-miR-16-5p | 0.001871293 | 0.018489609 |
| E3 ubiquitin ligases ubiquitinate target proteins | 61 | 19 | 2 | hsa-miR-15a-5p,hsa-miR-16-5p | 0.001871293 | 0.018489609 |
| Mitotic Prometaphase | 224 | 52 | 2 | hsa-miR-15a-5p,hsa-miR-16-5p | 0.001917474 | 0.018791243 |
| Antigen processing: Ubiquitination & Proteasome degradation | 336 | 73 | 2 | hsa-miR-15a-5p,hsa-miR-16-5p | 0.001912818 | 0.018791243 |
| Axon guidance | 603 | 121 | 2 | hsa-miR-15a-5p,hsa-miR-16-5p | 0.001953871 | 0.0190701 |
| PI Metabolism | 89 | 25 | 2 | hsa-miR-15a-5p,hsa-miR-16-5p | 0.002041268 | 0.019813441 |
| Metabolism of non-coding RNA | 57 | 18 | 2 | hsa-miR-15a-5p,hsa-miR-16-5p | 0.002054788 | 0.019813441 |
| snRNP Assembly | 57 | 18 | 2 | hsa-miR-15a-5p,hsa-miR-16-5p | 0.002054788 | 0.019813441 |
| DNA methylation | 66 | 20 | 2 | hsa-miR-15a-5p,hsa-miR-16-5p | 0.002082323 | 0.01999863 |
| Gene and protein expression by JAK-STAT signaling after Interleukin-12 stimulation | 40 | 14 | 2 | hsa-miR-15a-5p,hsa-miR-16-5p | 0.002150887 | 0.020574824 |
| Disassembly of the destruction complex and recruitment of AXIN to the membrane | 32 | 12 | 2 | hsa-miR-15a-5p,hsa-miR-16-5p | 0.002259552 | 0.021275232 |
| COPI-independent Golgi-to-ER retrograde traffic | 53 | 17 | 2 | hsa-miR-15a-5p,hsa-miR-16-5p | 0.002245061 | 0.021275232 |
| Defective TPR may confer susceptibility towards thyroid papillary carcinoma (TPC) | 32 | 12 | 2 | hsa-miR-15a-5p,hsa-miR-16-5p | 0.002259552 | 0.021275232 |
| Regulation of Glucokinase by Glucokinase Regulatory Protein | 32 | 12 | 2 | hsa-miR-15a-5p,hsa-miR-16-5p | 0.002259552 | 0.021275232 |
| Activation of BAD and translocation to mitochondria | 17 | 8 | 2 | hsa-miR-15a-5p,hsa-miR-16-5p | 0.002329126 | 0.021812096 |
| RNA Polymerase I Promoter Escape | 109 | 29 | 2 | hsa-miR-15a-5p,hsa-miR-16-5p | 0.002334739 | 0.021812096 |
| PKMTs methylate histone lysines | 76 | 22 | 2 | hsa-miR-15a-5p,hsa-miR-16-5p | 0.002445766 | 0.022672912 |
| RUNX1 interacts with co-factors whose precise effect on RUNX1 targets is not known | 49 | 16 | 2 | hsa-miR-15a-5p,hsa-miR-16-5p | 0.002437825 | 0.022672912 |
| EPH-Ephrin signaling | 95 | 26 | 2 | hsa-miR-15a-5p,hsa-miR-16-5p | 0.002507862 | 0.023159137 |
| Signaling by BRAF and RAF fusions | 67 | 20 | 2 | hsa-miR-15a-5p,hsa-miR-16-5p | 0.002538593 | 0.023353107 |
| RMTs methylate histone arginines | 81 | 23 | 2 | hsa-miR-15a-5p,hsa-miR-16-5p | 0.002597251 | 0.023801529 |
| Mitotic G1 phase and G1/S transition | 170 | 41 | 2 | hsa-miR-15a-5p,hsa-miR-16-5p | 0.002647991 | 0.024174241 |
| SARS-CoV-2 Infection | 72 | 21 | 2 | hsa-miR-15a-5p,hsa-miR-16-5p | 0.002744791 | 0.024963046 |
| Pre-NOTCH Processing in Golgi | 21 | 9 | 2 | hsa-miR-15a-5p,hsa-miR-16-5p | 0.002764382 | 0.025046342 |
| Potential therapeutics for SARS | 91 | 25 | 2 | hsa-miR-15a-5p,hsa-miR-16-5p | 0.002841727 | 0.025650323 |
| Golgi Cisternae Pericentriolar Stack Reorganization | 14 | 7 | 2 | hsa-miR-15a-5p,hsa-miR-16-5p | 0.002873587 | 0.025744333 |
| Chk1/Chk2(Cds1) mediated inactivation of Cyclin B:Cdk1 complex | 14 | 7 | 2 | hsa-miR-15a-5p,hsa-miR-16-5p | 0.002873587 | 0.025744333 |
| Cytosolic sensors of pathogen-associated DNA | 68 | 20 | 2 | hsa-miR-15a-5p,hsa-miR-16-5p | 0.003075992 | 0.027276955 |
| NOTCH1 Intracellular Domain Regulates Transcription | 50 | 16 | 2 | hsa-miR-15a-5p,hsa-miR-16-5p | 0.003071775 | 0.027276955 |
| RNA Polymerase III Transcription Initiation From Type 2 Promoter | 29 | 11 | 2 | hsa-miR-15a-5p,hsa-miR-16-5p | 0.00307874 | 0.027276955 |
| Host Interactions of HIV factors | 151 | 37 | 2 | hsa-miR-15a-5p,hsa-miR-16-5p | 0.003125876 | 0.027592748 |
| MET activates RAP1 and RAC1 | 11 | 6 | 2 | hsa-miR-15a-5p,hsa-miR-16-5p | 0.003358089 | 0.029426176 |
| Formation of annular gap junctions | 11 | 6 | 2 | hsa-miR-15a-5p,hsa-miR-16-5p | 0.003358089 | 0.029426176 |
| Neutrophil degranulation | 573 | 114 | 2 | hsa-miR-15a-5p,hsa-miR-16-5p | 0.003485274 | 0.030319358 |
| Nucleotide-binding domain, leucine rich repeat containing receptor (NLR) signaling pathways | 55 | 17 | 2 | hsa-miR-15a-5p,hsa-miR-16-5p | 0.003478228 | 0.030319358 |
| Inactivation of CDC42 and RAC1 | 8 | 5 | 2 | hsa-miR-15a-5p,hsa-miR-16-5p | 0.003500064 | 0.030338097 |
| RAF/MAP kinase cascade | 306 | 66 | 2 | hsa-miR-15a-5p,hsa-miR-16-5p | 0.003675896 | 0.031747574 |
| Cytokine Signaling in Immune system | 1050 | 196 | 2 | hsa-miR-15a-5p,hsa-miR-16-5p | 0.003762459 | 0.032378724 |
| TNF signaling | 51 | 16 | 2 | hsa-miR-15a-5p,hsa-miR-16-5p | 0.003835861 | 0.032825124 |
| MAPK1/MAPK3 signaling | 312 | 67 | 2 | hsa-miR-15a-5p,hsa-miR-16-5p | 0.003841674 | 0.032825124 |
| Signaling by plasma membrane FGFR1 fusions | 3 | 3 | 2 | hsa-miR-15a-5p,hsa-miR-16-5p | 0.003863199 | 0.032891987 |
| Translocation of SLC2A4 (GLUT4) to the plasma membrane | 74 | 21 | 2 | hsa-miR-15a-5p,hsa-miR-16-5p | 0.003933908 | 0.033375668 |
| TGF-beta receptor signaling activates SMADs | 34 | 12 | 2 | hsa-miR-15a-5p,hsa-miR-16-5p | 0.004051985 | 0.034256397 |
| Regulation of Insulin-like Growth Factor (IGF) transport and uptake by Insulin-like Growth Factor Binding Proteins (IGFBPs) | 128 | 32 | 2 | hsa-miR-15a-5p,hsa-miR-16-5p | 0.004142129 | 0.034895618 |
| RNA Polymerase III Transcription Initiation From Type 1 Promoter | 30 | 11 | 2 | hsa-miR-15a-5p,hsa-miR-16-5p | 0.004181881 | 0.035101654 |
| Cyclin A/B1/B2 associated events during G2/M transition | 26 | 10 | 2 | hsa-miR-15a-5p,hsa-miR-16-5p | 0.004195825 | 0.035101654 |
| Opioid Signalling | 94 | 25 | 2 | hsa-miR-15a-5p,hsa-miR-16-5p | 0.004530629 | 0.037770971 |
| RNA Polymerase III Abortive And Retractive Initiation | 43 | 14 | 2 | hsa-miR-15a-5p,hsa-miR-16-5p | 0.004588139 | 0.037856089 |
| RNA Polymerase III Transcription | 43 | 14 | 2 | hsa-miR-15a-5p,hsa-miR-16-5p | 0.004588139 | 0.037856089 |
| Transport of Mature mRNA Derived from an Intronless Transcript | 43 | 14 | 2 | hsa-miR-15a-5p,hsa-miR-16-5p | 0.004588139 | 0.037856089 |
| NoRC negatively regulates rRNA expression | 124 | 31 | 2 | hsa-miR-15a-5p,hsa-miR-16-5p | 0.004722248 | 0.038829167 |
| Ovarian tumor domain proteases | 39 | 13 | 2 | hsa-miR-15a-5p,hsa-miR-16-5p | 0.00495944 | 0.040502097 |
| Signaling by FGFR1 in disease | 39 | 13 | 2 | hsa-miR-15a-5p,hsa-miR-16-5p | 0.00495944 | 0.040502097 |
| Signalling to ERKs | 35 | 12 | 2 | hsa-miR-15a-5p,hsa-miR-16-5p | 0.005300502 | 0.042994948 |
| Transport of the SLBP independent Mature mRNA | 35 | 12 | 2 | hsa-miR-15a-5p,hsa-miR-16-5p | 0.005300502 | 0.042994948 |
| Transcription of E2F targets under negative control by DREAM complex | 19 | 8 | 2 | hsa-miR-15a-5p,hsa-miR-16-5p | 0.005403681 | 0.0436843 |
| Metal ion SLC transporters | 31 | 11 | 2 | hsa-miR-15a-5p,hsa-miR-16-5p | 0.005577695 | 0.044789449 |
| FGFR1 mutant receptor activation | 31 | 11 | 2 | hsa-miR-15a-5p,hsa-miR-16-5p | 0.005577695 | 0.044789449 |
| Macroautophagy | 146 | 35 | 2 | hsa-miR-15a-5p,hsa-miR-16-5p | 0.005759033 | 0.046073725 |
| Transport of Mature mRNAs Derived from Intronless Transcripts | 44 | 14 | 2 | hsa-miR-15a-5p,hsa-miR-16-5p | 0.005776006 | 0.046073725 |
| VLDLR internalisation and degradation | 12 | 6 | 2 | hsa-miR-15a-5p,hsa-miR-16-5p | 0.005829962 | 0.046197158 |
| Gap junction degradation | 12 | 6 | 2 | hsa-miR-15a-5p,hsa-miR-16-5p | 0.005829962 | 0.046197158 |
| Beta-catenin independent WNT signaling | 167 | 39 | 2 | hsa-miR-15a-5p,hsa-miR-16-5p | 0.005935845 | 0.046881458 |
| FOXO-mediated transcription | 67 | 19 | 2 | hsa-miR-15a-5p,hsa-miR-16-5p | 0.005977321 | 0.047054256 |
| Amyloid fiber formation | 111 | 28 | 2 | hsa-miR-15a-5p,hsa-miR-16-5p | 0.006157801 | 0.048316604 |
| NGF-stimulated transcription | 40 | 13 | 2 | hsa-miR-15a-5p,hsa-miR-16-5p | 0.006311707 | 0.049362891 |
